# Microbiome analysis reveals universal diagnostic biomarkers for colorectal cancer across populations and technologies

**DOI:** 10.3389/fmicb.2022.1005201

**Published:** 2022-11-03

**Authors:** Huarong Zhang, Junling Wu, Daihan Ji, Yijuan Liu, Shuting Lu, Zeman Lin, Ting Chen, Lu Ao

**Affiliations:** ^1^Key Laboratory of Ministry of Education for Gastrointestinal Cancer, The School of Basic Medical Sciences, Fujian Medical University, Fuzhou, China; ^2^Fujian Key Laboratory of Medical Bioinformatics, Department of Bioinformatics, School of Medical Technology and Engineering, Fujian Medical University, Fuzhou, China; ^3^Department of Gastroenterology, The First Affiliated Hospital of Fujian Medical University, Fuzhou, China

**Keywords:** microbial dysbiosis, *Porphyromonas*, *Parvimonas*, *Peptostreptococcus*, non-invasive diagnosis, colorectal cancer

## Abstract

The gut microbial dysbiosis is a risk of colorectal cancer (CRC) and some bacteria have been reported as potential markers for CRC diagnosis. However, heterogeneity among studies with different populations and technologies lead to inconsistent results. Here, we investigated six metagenomic profiles of stool samples from healthy controls (HC), colorectal adenoma (CA) and CRC, and six and four genera were consistently altered between CRC and HC or CA across populations, respectively. In FengQ cohort, which composed with 61 HC, 47 CA, and 46 CRC samples, a random forest (RF) model composed of the six genera, denoted as signature-HC, distinguished CRC from HC with an area under the curve (AUC) of 0.84. Similarly, another RF model composed of the four universal genera, denoted as signature-CA, discriminated CRC from CA with an AUC of 0.73. These signatures were further validated in five metagenomic sequencing cohorts and six independent 16S rRNA gene sequencing cohorts. Interestingly, three genera overlapped in the two models (*Porphyromonas*, *Parvimonas* and *Peptostreptococcus*) were with very low abundance in HC and CA, but sharply increased in CRC. A concise RF model on the three genera distinguished CRC from HC or CA with AUC of 0.87 and 0.67, respectively. Functional gene family analysis revealed that Kyoto Encyclopedia of Genes and Genomes Orthogroups categories which were significantly correlated with markers in signature-HC and signature-CA were mapped into pathways related to lipopolysaccharide and sulfur metabolism, which might be vital risk factors of CRC development. Conclusively, our study identified universal bacterial markers across populations and technologies as potential aids in non-invasive diagnosis of CRC.

## Introduction

Colorectal cancer (CRC) is a heterogeneous disease of the intestinal epithelium, which is one of the leading causes of cancer death worldwide. The causes of CRC are complex and varied. It has been shown that genetic factors account for only 10%–30% of the CRC risk and environmental factors play a significant role in causing CRC (Jasperson et al., 2010; Jie et al., 2017; Manson et al., 2019). Human gut microbiota, which contains trillions of microorganisms, has been confirmed as an important environmental carcinogenic factor. Most CRCs develop from normal epithelium to colorectal adenoma (CA), and further develop into malignant tumors through accumulation of abnormal changes in oncogenes and tumor suppressor genes, and persistent imbalance of intestinal flora (Fearon and Vogelstein, 1990). Many studies have demonstrated that CRC patients are accompanied by gut microbiota dysbiosis (Zackular et al., 2014; Feng et al., 2015; Liang et al., 2020).

Meanwhile, the survival rate gradually decreases with the progress of CRC. According to the American Joint Committee on Cancer (AJCC), the 5-year survival rate of early CRC (stage II and below) is about 90%, while it is less than 10% for stage IV CRC patients. Hence the early diagnosis of CRC is crucial for the improvement of prognosis. Colonoscopy, a population-wide screening and prevention program, which is applied in many countries, needs many preoperative preparations and high cost. Fecal occult blood testing (FOBT) and carcinoembryonic antigen (CEA), currently the standard noninvasive screening tests (Levin et al., 2008; Zavoral et al., 2009), have limited sensitivity and specificity for CRC (Krzystek-Korpacka et al., 2013). Using fecal microbiota in CRC screening can serve as a non-invasive complement for early diagnosis of CRC.

Based on 16S rRNA gene sequencing and metagenome technologies, many fecal microbial markers of CRC have been identified (Feng et al., 2015; Yu et al., 2017; Chung et al., 2018; Liang et al., 2020; Lowenmark et al., 2020; Zhang et al., 2020). However, the reproducibility and the predictive accuracy of these microbial markers across cohorts remains unclear. Specially, the microbial compositions of samples vary greatly among populations (Feng et al., 2015; Yu et al., 2017; Dadkhah et al., 2019; Zhang et al., 2020). Some studies have revealed cross-cohort microbial markers for CRC diagnosis using either metagenomic sequencing or 16S rRNA gene sequencing (Dai et al., 2018; Thomas et al., 2019; Wirbel et al., 2019; Wu et al., 2021b). Nevertheless, there are few microbial markers can be applied into both the two technologies. Therefore, it is urgent to identify universal microbial markers for early diagnosis of CRC, which are insusceptible to geographic and technical differences.

In this work, we analyzed 705 fecal samples in six metagenomic sequencing cohorts and 799 samples in six 16S rRNA cohorts, which were collected from different regions and ethnics. Six and four genera were significantly different between CRC and healthy controls (HC) or CA group, respectively. Random forest (RF) models for distinguishing CRC from HC or CA group, denoted as signature-HC and signature-CA, respectively, achieved high accuracies (CRC versus HC: AUC = 0.84; CRC versus CA: AUC = 0.73) in the training cohort. The two RF models were further validated in five metagenomic cohorts and six 16S rRNA cohorts. Moreover, the two models were specific to CRC against other metabolic diseases, including type 2 diabetes (T2D), obese, ulcerative colitis (UC) and Crohn’s disease (CD). Importantly, three genera (*Porphyromonas*, *Parvimonas* and *Peptostreptococcus*), which were overlapped in the two signatures, were with very low abundance in HC and CA, but sharply increased in CRC. A concise RF models on these three genera also distinguished CRC from HC or CA group with high accuracies. According to functional gene family analysis, 103 CRC-associated Kyoto Encyclopedia of Genes and Genomes Orthogroups (KO) categories which were significantly correlated with genera in signature-HC or signature-CA were mapped to malignancy-associated pathways. These results have proven the validity of CRC-specific markers across populations and technologies, which would be an auxiliary method for non-invasive diagnosis.

## Materials and methods

### Sample characteristics and data preprocessing

Six public fecal metagenomic CRC datasets (Zeller et al., 2014; Feng et al., 2015; Vogtmann et al., 2016; Yu et al., 2017; Thomas et al., 2019) collected from Australia, China, France, Italy, Germany and United States were directly downloaded from curatedMetagenomicData Rpackage (Pasolli et al., 2017; accessed on September 9, 2021), and the taxonomic profiles were retrieved using the keyword “*metaphlan_bugs_list.stool*.” The MetaPhlAn2 (Truong et al., 2015) pipeline was used to infer the taxonomic abundance profiles from the presence and read coverage of clade-specific markers in microbiome samples. The HUMAnN2 (Abubucker et al., 2012) pipeline was used to define the pathway abundance profiles which were presented as the average abundance of the top 50% genes with high abundance in the pathway. Totally, 324 CRC, 116 CA, and 285 HC were included (Table 1). Meanwhile, six 16S rRNA gene sequencing datasets (Zackular et al., 2014; Zeller et al., 2014; Nakatsu et al., 2015; Baxter et al., 2016; Deng et al., 2018; Yang et al., 2019) composing of 205 CRC, 301 CA, and 293 HC mainly from Canada, China, France and United States were downloaded from Sequence Read Archive (SRA),^1^ and Microbiome Biomarker CRC.^2^ Each the 16S rRNA gene sequencing dataset was uniformly processed as follows (Supplementary Figure S1): (i) downloaded the raw sequencing data; (ii) filtered out sequencing reads with quality score *Q* > 25 and denoise reads into operational taxonomic units (OTUs; i.e., 97% exact sequence match) by VSEARCH (Rognes et al., 2016), which was wrapped in the Quantitative Insights Into Microbial Ecology 2 (QIIME2; Caporaso et al., 2010); (iii) assigned taxonomy classification based on the naive Bayes classifier using the classify-sklearn package against the Silva reference database^3^ (Quast et al., 2013). In order to apply our proposed bacterial biomarkers to both the metagenomic and 16S rRNA gene sequencing data, all taxonomic profiles at the genus level were used for subsequent analysis.

**Table 1:** The demographic information of CRC datasets.

Dataset	Group (Number)	Age (Mean)	BMI (Mean)	Sex (F%/M%)	Technology	Country
FengQ	HC (61)	66.97	27.61	59.02/40.98	Shotgun	Australia
	CA (47)	66.49	27.96	48.94/51.06		
	CRC (46)	67.07	26.50	50.00/50.00		
ZellerG	HC (66)	58.77	24.72	50.00/50.00	Shotgun	France/Germany
	CA (42)	62.95	25.90	71.43/28.54		
	CRC (91)	64.66	26.05	59.34/40.66		
YuJ	HC (54)	NA	NA	NA	Shotgun	China
	CRC (74)	NA	NA	NA		
ThomasAM_2018a	HC (24)	67.92	25.32	54.17/45.83	Shotgun	Italy
	CA (27)	62.89	28.00	59.26/40.74		
	CRC (29)	71.45	25.71	79.31/20.69		
ThomasAM_2018b	HC (28)	NA	NA	NA	Shotgun	Italy
	CRC (32)	NA	NA	NA		
VogtmannE	HC (52)	63.23	25.35	71.15/28.85	Shotgun	United States
	CRC (52)	61.85	24.89	71.15/28.85		
PRJNA464414	HC (33)	NA	NA	NA	16S rRNA	China
	CRC (17)	NA	NA	NA		
PRJEB6070	HC (50)	62.32	24.66	48.00/52.00	16S rRNA	France
	CA (38)	62.29	24.95	68.42/31.58		
	CRC (41)	65.51	25.44	33.80/66.20		
PRJNA280026	HC (61)	NA	NA	NA	16S rRNA	China
	CA (57)	NA	NA	NA		
	CRC (52)	NA	NA	NA		
PRJNA290926	CA (48)	62.1	26.4	33.3/66.7	16S rRNA	United States
	CRC (29)	60.3	28.3	48.3/51.7		
Zackular	HC (30)	55.3	26.6	63/37	16S rRNA	Canada/United States
	CA (30)	61.3	27.4	40/60		
	CRC (30)	59.4	30.7	30/70		
PRJNA430990	HC (119)	NA	NA	NA	16S rRNA	China
	CA (128)	NA	NA	NA		
	CRC (36)	NA	NA	NA		

Moreover, to identify the differential genera in all samples mixed from the six metagenomics datasets, de-batch processing by the MMUPHin Rpackage (Ma et al., 2020) was performed to avoid biases from different studies due to different taxonomic classifiers, reference databases, etc.

### Microbial community analysis

The microbial community analysis, including α-diversity and β-diversity, were calculated by “vegan” Rpackage (Oksanen et al., 2020). The α-diversity was evaluated by Shannon index and richness. Kruskal-Wallis rank-sum test was used for differential test of α-diversity. Bray–Curtis distance of β-diversity was used for principal coordinate analysis (PCoA) and community discrepancy was test by permutational multivariate analyses of variance (PERMANOVA) with 999 permutations. Betadisper test was used to assess the degree of dispersion in different datasets.

### Confounder analysis

Analysis of Variance (ANOVA) was used to quantify the effect of potential confounding factors relative to that of CRC on a single microbial species. The total variance within the abundance of a given microbial species was compared to the variance explained by disease status and the variance explained by the confounding factor using a linear model. Body mass index (BMI), one of potential confounders, with continuous values was converted to categorical data of lean/normal/overweight/obese according to conventional cutoffs (thin: <20; normal: 20–25; overweight: 25–30; obese: >30).

### Model construction and feature extraction

A two-sided Wilcoxon rank-sum test for microbes with differential abundance were applied at the phylum, genus and species levels, respectively. The direction of dysregulation was determined by the difference between the means of different groups. Then, the common different flora which had consistent dysregulated direction in three or two metagenomic datasets were extracted as the important features to construct RF models for distinguishing CRC from HC or CA, respectively. The training dataset FengQ was used to set two parameters of RF models (mtry and ntree), and the out-of-bag error rate was taken as a reference to determine the optimal combination. The AUC, accuracy, sensitivity, specificity and F-score were used to evaluate the performances of RF models. F-score was calculated as follow:


$$F-\mathrm{score}=2\times \frac{\mathrm{Precision}\times \mathrm{Recall}}{\mathrm{Precision}+\mathrm{Recall}}$$

To assess the generalizability of microbial-based classifiers across samples with geographic and technical differences, both study-to-study transfer validation and leave-one-dataset-out (LODO) validation were performed. In study-to-study transfer validation, features were trained in one single cohort and assessed on all other cohorts. In LODO validation, one cohort was set as the test set, and the other cohorts were combined into the training set. A *p* < 0.05 was considered as significant unless otherwise stated.

### Functional gene family analysis

Gene families determined by UniRef90 were mapped to KO database and were grouped into functional categories by HMP Unified Metabolic Analysis Network (HUMAnN3; Franzosa et al., 2018). The conditioned two-sided Wilcoxon rank-sum test was used to estimate *p*-value for the abundance change of the KO categories from HC or CA to CRC in each cohort. Then, the common different KO categories which had consistent dysregulation direction in multiple metagenomic datasets were identified as the CRC-associated KO categories. Spearman correlation was applied to estimate the relationship between bacterial markers in signature-HC and signature-CA and CRC-associated KO categories. All statistical analyses were done by using the R 3.6.3.

## Results

### Overview of the microbial metagenomic profiles

First, microbiota diversity and microbial composition was carried out on a study-by-study. Kruskal-Wallis rank-sum test showed that significant differences in the richness were observed between the CRC and HC groups in four metagenomic sequencing datasets (FengQ, *p* = 4.80e−08; ThomasAM_2018b, *p* = 2.00e−03; VogtmannE, *p* = 0.01; YuJ, *p* = 1.00e−03, Supplementary Figure S2A). Comparing to the HC group, the richness was increasing in the CRC group. The Shannon index of α-diversity showed no significant difference between groups, while CRC increased slightly and reached to the edge of a significant level in FengQ (*p* = 9.10e−02, Supplementary Figure S2B). To display microbiome space between samples, β-diversity based on Bray–Curtis distance was calculated by PERMANOVA. The results showed that the fecal microbiota compositions among groups were significantly different in ZellerG and YuJ (*p* = 1.00e−03, Supplementary Figure S3), moderately different in FengQ (*p* = 6.90e−02), and could be separated in VogtmannE (*p* = 0.12) and ThomasAM_2018a (*p* = 0.11).

**Figure 1:**
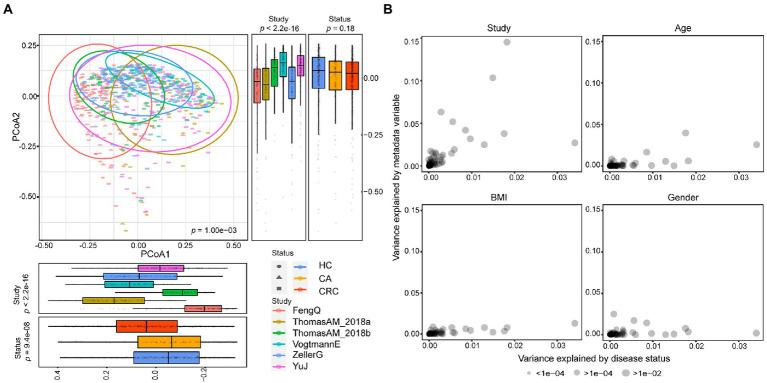
Comparison of heterogeneity between different studies. **(A)** A strong influence of study heterogeneity on beta diversity. PCoA analysis of samples from six metagenomic sequencing studies based on Bray–Curtis distance showed the fecal microbiota composition was different among studies (*p* < 0.001) and groups (*p* < 0.001). Studies were color-coded and groups (HC, CA, and CRC) were indicated by different shapes. The upper-right and the bottom-left boxplots illustrated that samples were projected onto the first two principal coordinates broken down by study and disease status, respectively. **(B)** The associations of potential confounding factors of individual microbial species with patient geographic and technical factors. Variances explained by disease status (CRC versus CA or HC) were plotted against variances explained by different confounding factors, including age, gender and BMI. Each species was represented by a dot proportional in size to its abundance; For the confounder analysis, the BMI was split into lean/normal/overweight/obese according to cutoffs (thin: <20; normal: 20–25; overweight: 25–30; obese: >30).

Then, all samples in the six fecal metagenomic CRC datasets were pooled together to perform PCoA analysis to examine the biological and technical differences in across-cohorts. As shown in Figure 1A, the PERMANOVA test showed that the microbiota compositions among studies were significantly different (*p* = 1.00e−03). We further evaluated whether the difference was affected by the degree of dispersion in different datasets. The result showed that the center points of six datasets were very close in the space defined by PCoA, but the degree of dispersion of samples in different datasets was significantly different (betadisper test: *p* = 1.00e−03, Supplementary Figure S4). The results indicated that the spatial distribution of samples was the main reason for the remarkable results of PERMANOVA.

Meanwhile, we observed samples from the same study tended to cluster together. Different studies showed more significant statistical differences in PCoA1 and PCoA2 than that in different disease status, suggesting that the difference between datasets was greater than that between the disease status. In another word, study heterogeneity had a strong effect on β-diversity (Figure 1A), which was consistent with previous studies (Thomas et al., 2019; Wu et al., 2021b). Furthermore, the effect of study heterogeneity on microbiome composition was quantified and compared with other potential confounding factors (patient age, BMI, gender). The result also demonstrated that compared to other confounding factors, study heterogeneity was the main factor affecting the composition of microbial species (Figure 1B). Therefore, the subsequent analysis was separately performed in each dataset.

### Alterations of the taxonomic composition in CRC

**Figure 2:**
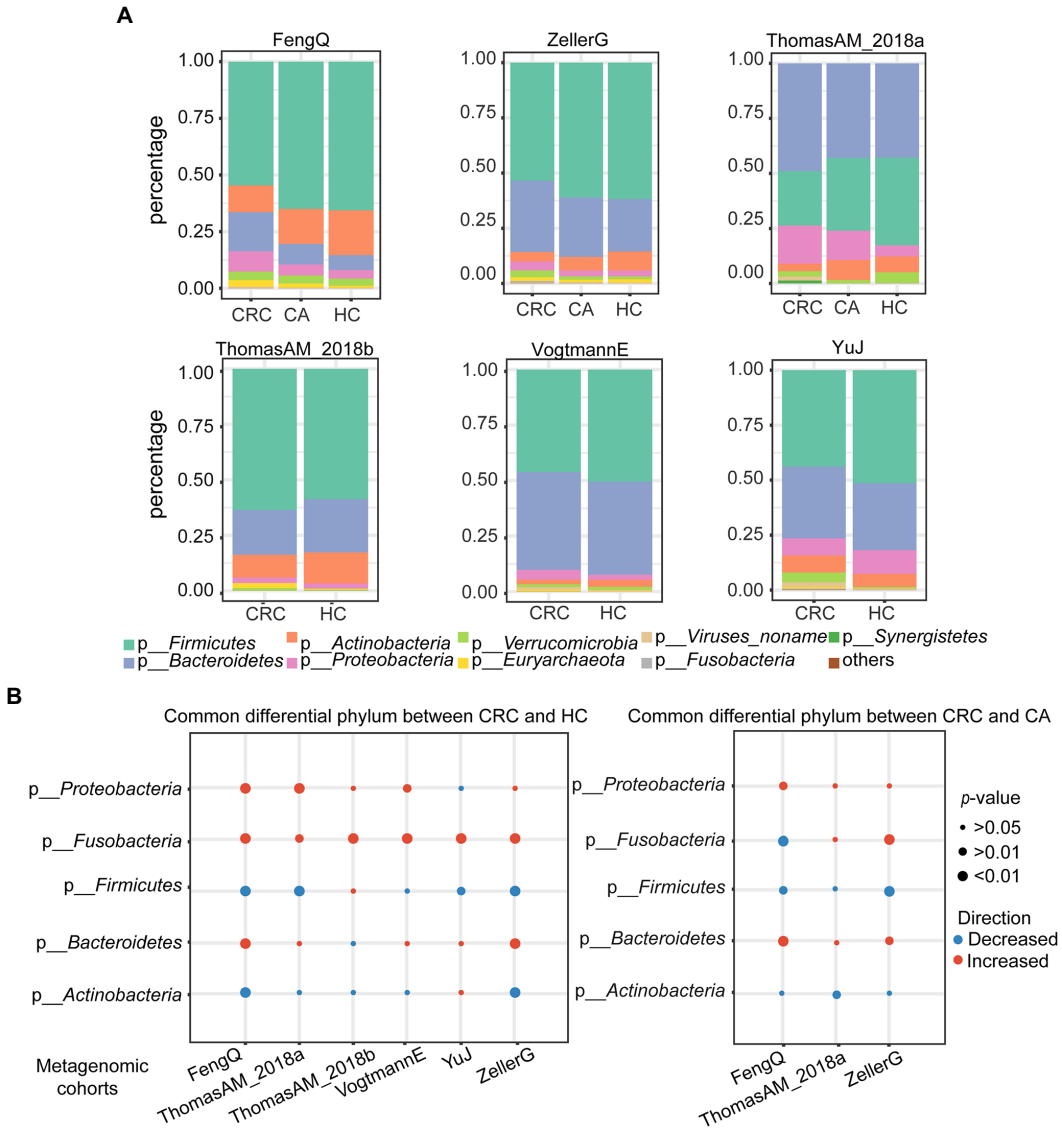
Principal and significantly differential microbes at phylum level in six metagenomic sequencing datasets. **(A)** Relative proportions of top eight principal phyla in HC, CA, and CRC. **(B)** Alterations of top five phyla between CRC and HC (left) or CA (right). Red and blue indicated phyla were increased and decreased in the CRC group, respectively. *p*-value was calculated by Wilcoxon rank-sum test.

To find the universal differential microbes for CRC diagnosis across cohorts, significant differential bacteria between CRC and HC or CA were identified at the phylum, genus and species levels, respectively. At the phylum level, the gut microbiota in HC, CA and CRC were all dominated by members of *Firmicutes*, *Bacteroidetes*, *Actinobacteria*, *Proteobacteria*, *Fusobacteria* and *Verrucomicrobia* (Figure 2A). No significant difference was observed in the composition of the flora among the groups in six metagenomic datasets (chi-square test, FengQ: *p* = 0.66, ZellerG: *p* = 0.98, ThomasAM_2018a: *p* = 0.18, ThomasAM_2018b: *p* = 0.95, VogtmannE: *p* = 0.99 and YuJ: *p* = 0.64). The phylum *Proteobacteria* and *Fusobacteria* had significantly increased abundance in CRC versus HC, while the phylum *Firmicutes* had significantly decreased abundance in CRC compared to that in HC and CA groups (Wilcoxon rank-sum test, *p* < 0.05, Figure 2B; Supplementary Tables S1, Supplementary TableS2).

**Figure 3:**
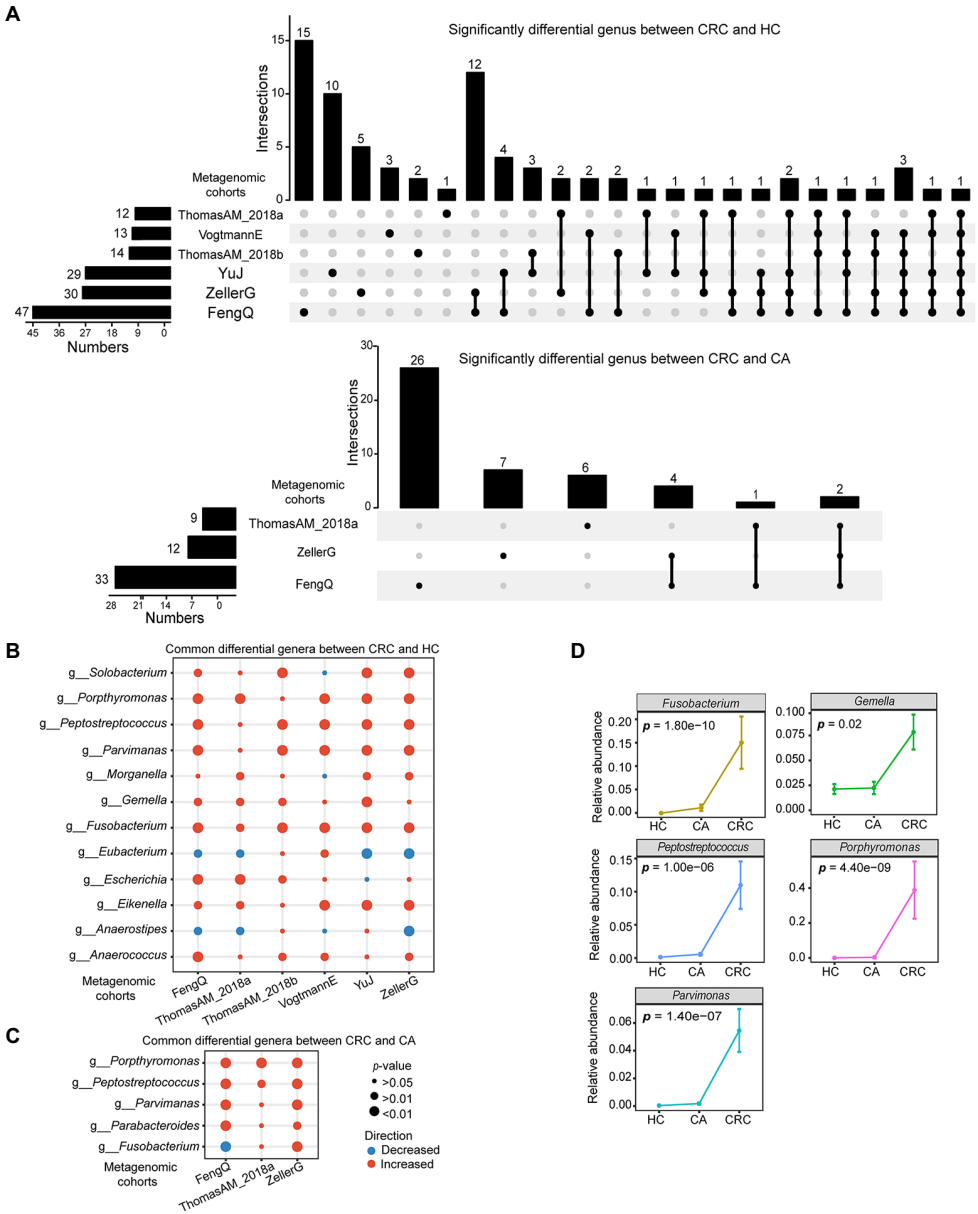
Significantly differential microbes at genus level in six metagenomic sequencing datasets. **(A)** UpSet plot showed significantly differential genus between CRC and HC or CA. **(B,C)** Bubble plot showed universal differential genus between CRC and HC or CA. **(D)** Five genera that abundances were significantly increased with disease progressed at FengQ cohort. *p*-value was calculated by Wilcoxon rank-sum test.

At genus level, there were 117 genera commonly detected in six cohorts. Using Wilcoxon rank-sum test with *p* < 0.05, 47, 30, 29, 14, 13, and 12 differential genera were identified between CRC and HC groups in FengQ, ZellerG, YuJ, ThomasAM_2018b, VogtmannE and ThomasAM_2018a, respectively, of which 36 genera were cohort specific, while 12 universal genera were consistently dysregulated in at least three cohorts (Figure 3A). Compared with HC, *Anaerostipes* and *Eubacterium* were significantly decreased in CRC, while *Porphyromonas, Fusobacterium, Parvimonas, Peptostreptococcus, Gemella, Eikenella, Escherichia, Anaerococcus, Solobacterium* and *Morganella* were significantly increased (Figure 3B; Supplementary Table S3). Similarly, 33, 13, and 9 differential genera were identified between CRC and CA groups in FengQ, ZellerG and ThomasAM_2018a, of which 39 genera were cohort specific, while 4 universal genera (*Parabacteroides, Parvimonas, Peptostreptococcus* and *Porphyromanas*) were consistently dysregulated in at least two cohorts (Figures 3A,C; Supplementary Table S4). Moreover, *Fusobacterium, Parvimonas, Peptostreptococcus, Gemella* and *Porphyromanas* were significantly increased as disease progressed in FengQ (Figure 3D). Similar results were observed in dataset ZellerG and ThomasAM_2018a (Supplementary Figure S5).

**Figure 4:**
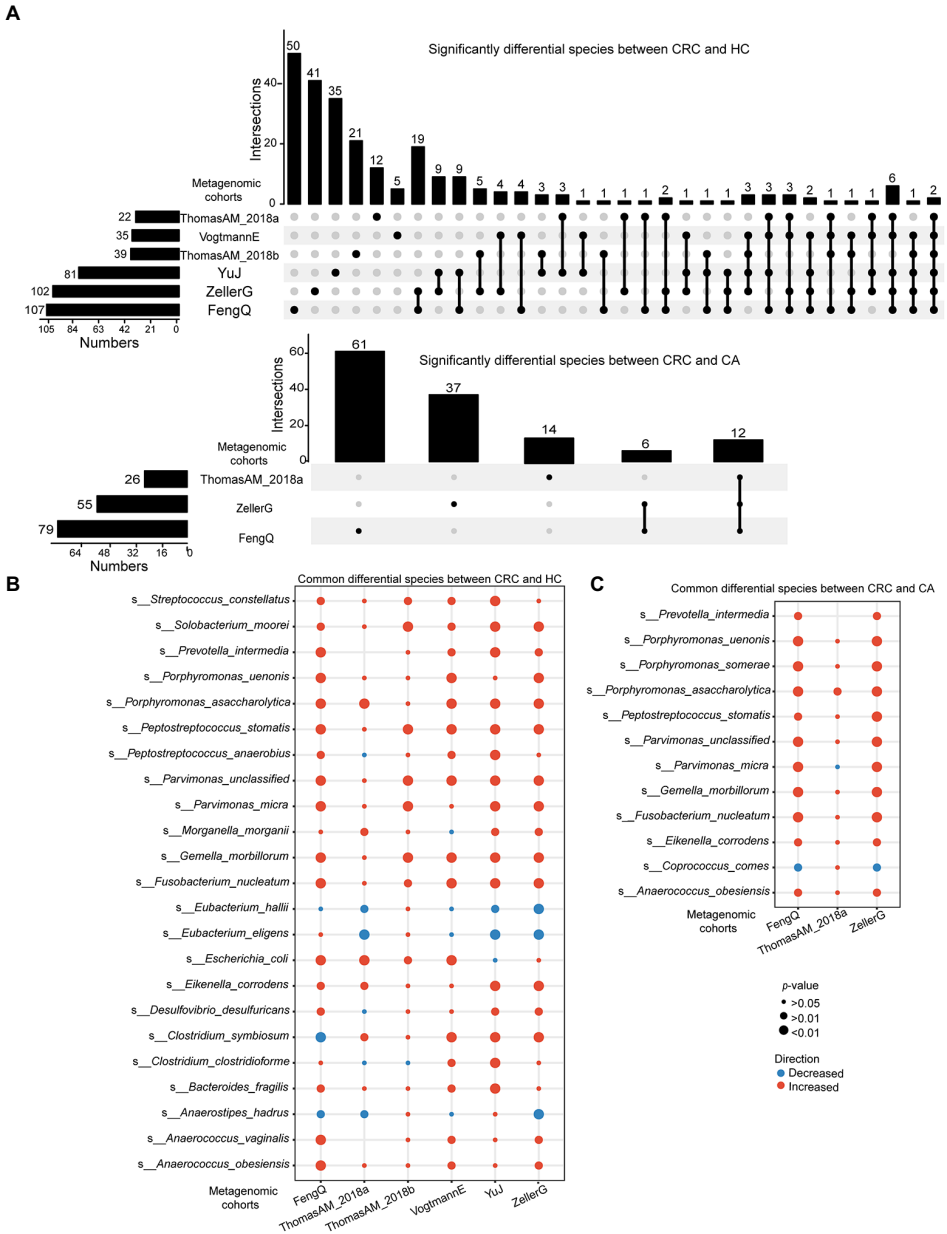
Significantly differential microbes at species level in six metagenomic sequencing datasets. **(A)** UpSet plot showed significantly differential species between CRC and HC or CA. **(B,C)** Bubble plot showed universal differential species between CRC and HC or CA. *p*-value was calculated by Wilcoxon rank-sum test.

At species level, 107, 102, 81, 39, 35, and 22 species with distinguishable differential abundance were identified between CRC and HC groups in FengQ, ZellerG, YuJ, ThomasAM_2018b, VogtmannE and ThomasAM_2018a, respectively, of which 164 species were cohort-specific (Wilcoxon rank-sum test, *p* < 0.05, Figure 4A). Specially, 22 universal species belonging to 18 genera were consistently dysregulated in at least three cohorts (Figure 4B; Supplementary Table S5). *Anaerostipes hadrus*, *Eubacterium eligens* and *Eubacterium hallii* were significantly decreased in CRC compared to that in HC group, while the other 19 species, which were assigned as *Peptostreptococcus anaerobius*, *Porphyromonas uenonis*, *Fusobacterium nucleatum*, *Gemella morbillorum*, et., were significantly increased in CRC.

Similarly, 79, 26, and 55 species with distinguishable differential abundance were identified between CRC and CA groups in FengQ, ThomasAM_2018a and ZellerG, respectively, of which 122 species were cohort-specific (Wilcoxon rank-sum test, *p* < 0.05, Figure 4A; Supplementary Table S6). As shown in Figures 4C, 11 universal species belonging to 9 genera were consistently dysregulated in at least two cohorts. *Coprococcus comes* was significantly decreased in CRC compared to that in CA group, while the other 10 species, e.g., *Anaerococcus obesiensis*, *P. uenonis*, *F. nucleatum*, *G. morbillorum*, were significantly increased in CRC. Importantly, the pathogenic bacteria *F. nucleatum, Peptostreptococcus stomatis, Parvimonas micra, Eikenella corrodens* and *Porphyromonas asaccharolytica* were enriched in CRC group compared with HC and CA groups.

Moreover, the six metagenomic sequencing datasets were integrated together to investigate whether the universal differential microbes identified above can be obtained in the mixed data from six metagenomic cohorts with 324 CRC, 116 CA, and 285 HC samples. Using Wilcoxon rank-sum test with *p* < 0.05, 71 and 57 significantly differential genera were identified between CRC and HC or CA groups, respectively. Compared with HC, 23 genera, e.g., *Anaerostipes*, *Faecalibacterium*, *Coprococcus* and *Eubacterium*, were significantly decreased in CRC, while 48 genera, e.g., *Porphyromonas*, *Fusobacterium*, *Parvimonas*, *Peptostreptococcus*, *Gemella*, *Eikenella*, *Escherichia*, *Anaerococcus*, *Solobacterium* and *Morganella*, were significantly increased (Supplementary Table S7). Notably, 11 of 12 universal genera identified beforehand between CRC and HC were also presented in the mixed data, all of which had consistent dysregulated directions in the two types of analysis. Similarly, compared with CA, 24 genera were significantly decreased in CRC, while the other 33 genera were significantly increased (Supplementary Table S8). All the four universal genera identified beforehand between CRC and CA had consistent dysregulated directions in the mixed data. The results indicated that the universal differential bacteria separately identified from multiple datasets were consistently altered in CRC across different studies.

Conclusively, the microbial composition altered with disease progression and some microbiota with consistent alternations were observed across multiple cohorts.

### Identification of microbiological markers for CRC versus HC

**Figure 5:**
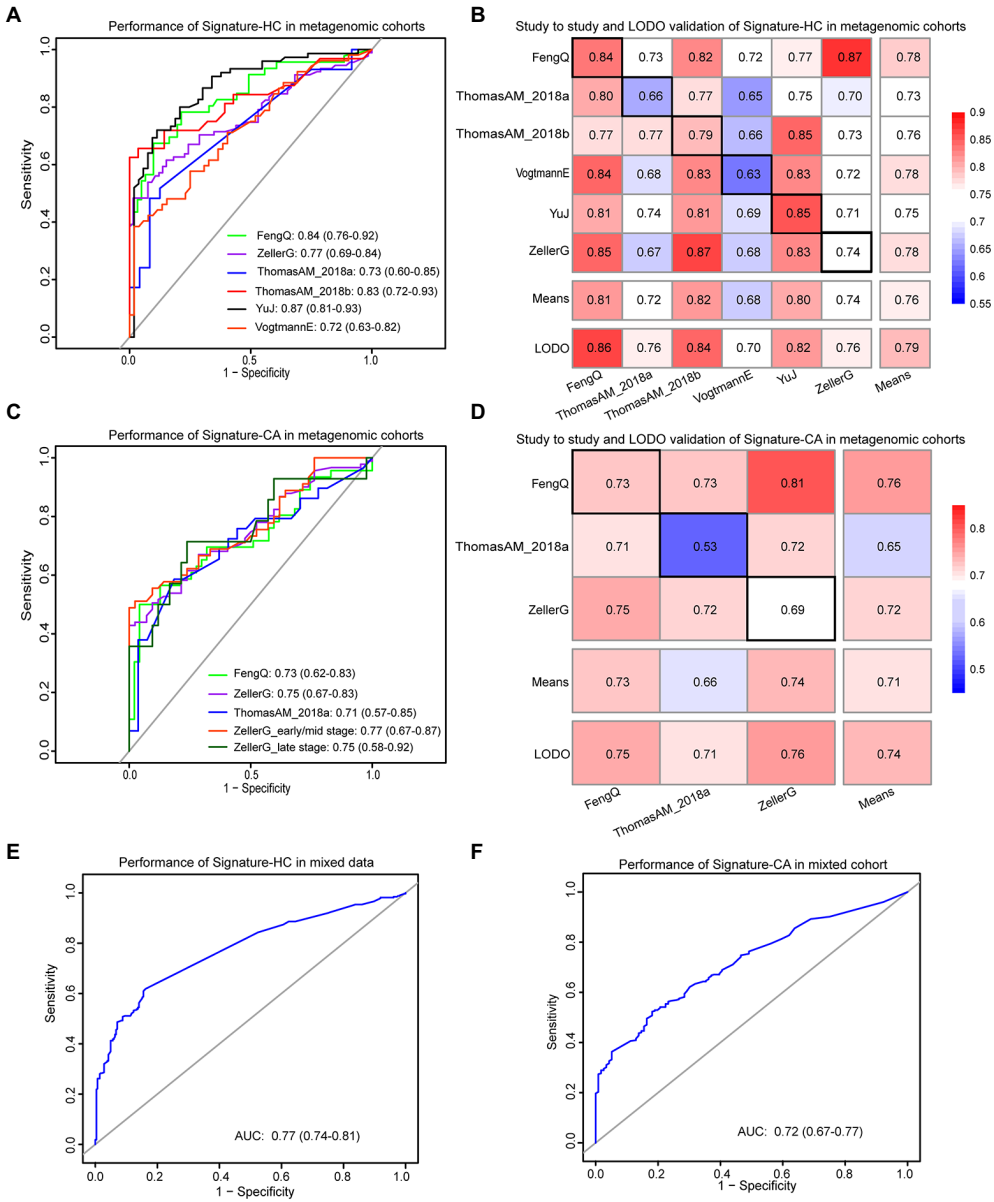
The performances of signature-HC and signature-CA across metagenomic data. **(A)** Performance of the signature-HC across six metagenomic sequencing cohorts. **(B)** The classification accuracy of signature-HC resulting from study-to-study transfer and LODO model. Values on the diagonal (black boxes) refer to the AUC values by training the model in the study of the corresponding row, while off-diagonal values refer to the AUC values by testing the model in the study of the corresponding column. The LODO rows refer to the AUC values obtained by the model on universal genera, using all but the study of the corresponding column and applying it to the study of the corresponding column. **(C)** Performance of the signature-CA across three metagenomic sequencing cohorts. **(D)** The classification accuracy of signature-CA resulting from study-to-study transfer and LODO model. **(E)** Performance of the signature-HC in the mixed data. **(F)** Performance of the signature-CA in the mixed data.

Among the 12 universal genera with distinguishable differential abundance between CRC and HC groups, six genera (*Anaerostipes*, *Porphyromonas*, *Fusobacterium*, *Parvimonas*, *Peptostreptococcus* and *Gemella*) commonly detected by 16S rRNA gene sequencing data were identified as important microbiological markers for CRC versus HC. They were belonged to three phyla, *Firmicutes*, *Bacteroidetes* and *Fusobacteria*. In the training dataset FengQ, a RF model based on the six genera with optimal parameter combination for mtry = 1 and ntree = 400, named signature-HC, achieved an AUC of 0.84 for distinguishing CRC from HC samples (95% CI: 0.76–0.92, accuracy: 0.78, sensitivity: 0.61, specificity: 0.90 and F-score: 0.70, Figure 5A). Then, signature-HC was tested at the other five metagenomic cohorts. The achieved AUCs between the CRC and HC groups in ThomasAM_2018a and ThomasAM_2018b were 0.73 and 0.83 with a 95% CI of 0.60–0.85 and 0.72–0.93, respectively. Similarly, the achieved AUCs in VogtmannE, ZellerG and YuJ datasets were 0.72 with a 95% CI of 0.63–0.82, 0.77 with a 95% CI of 0.69–0.93 and 0.87 with a 95% CI of 0.81–0.93, respectively.

In addition, both study-to-study validation and LODO validation were performed on the six metagenomic datasets (see Materials and methods for detail). As shown in Figure 5B, the AUC values ranged from 0.63 to 0.87, while the mean of AUC and the highest value was 0.76 and 0.87, respectively. Collectively, signature-HC could be applied to metagenomic data from different regions with different ethic, dietary intake and lifestyle.

### Identification of microbiological markers for CRC versus CA

Similarly, among the 11 universal genera with distinguishable differential abundance between CRC and CA groups, four genera (*Parabacteroides*, *Porphyromonas*, *Parvimonas* and *Peptostreptococcus*) commonly detected by 16S rRNA gene sequencing were used to construct a RF model to discriminate CRC from CA, named signature-CA. The RF model with optimal parameter combination for mtry = 1 and ntree = 400 achieved an AUC of 0.73 for distinguishing CRC from CA in FengQ cohort (95% CI: 0.62–0.83, accuracy: 0.72, sensitivity: 0.57, specificity: 0.87 and F-score: 0.67, Figure 5C). The achieved AUCs between the CRC and CA group in ZellerG and ThomasAM_2018a were 0.75 (95% CI: 0.67–0.83, accuracy: 0.68, sensitivity: 0.66, specificity: 0.71 and F-score: 0.74) and 0.71 (95% CI: 0.57–0.85, accuracy: 0.70, sensitivity: 0.59, specificity: 0.81 and F-score: 0.67). Moreover, the achieved AUC between stage I–II CRC and CA group in ZellerG was 0.77 (95% CI: 0.67–0.87, accuracy: 0.69, sensitivity: 0.67, specificity: 0.71 and F-score: 0.69). The performance was higher than CEA, which was with a 35% sensitivity for CRC patients (Chen et al., 2021) and a 52% accuracy for early-stage CRC patients (Krzystek-Korpacka et al., 2013). As shown in Figure 5D, the AUC values of study-to-study validation and LODO validation ranged from 0.53 to 0.81, while the mean and the highest AUC values was 0.74 and 0.81, respectively. These results indicated signature-CA could be applied to metagenomic data from different regions for early detection of CRC.

In the mixed data from the six metagenomic sequencing cohorts, the signature-HC and signature-CA achieved AUCs of 0.78 with a 95% CI of 0.74–0.81 (Figure 5E) and 0.72 with a 95% CI of 0.67–0.77 (Figure 5F), respectively. These results demonstrated that the two sets of universal microbiological markers were also robustly applied to the mixed data.

### Comparison of predictive performance using previous models

The performances of signature-HC and signature-CA were compared with four models proposed in publications presented the data. The LASSO logistic regression model proposed by Zeller et al. and the RF model proposed by Feng et al. performed well in their own data. We cannot further evaluate their performances in other datasets due to the lack of specific parameters for these models. Another two models proposed by Yu et al. and Thomas et al. were analyzed in multiple datasets, and compared with our signatures. As shown in Supplementary Figure S6A, the AUC values of signature-HC in most of testing datasets were higher than the two models. Meanwhile, the average AUC value of signature-HC in study-to-study validation was slightly higher than the model proposed by Thomas et al. (Supplementary Figure S6B). Model for discriminating CRC from CA was only proposed by Thomas et al., and the comparison also showed that the average AUC value of signature-CA was higher (Supplementary Figure S6C). These results indicated better performances of our signatures for discriminating CRC from HC or CA.

### Potential classification efficiency for 16S rRNA gene sequencing data

**Figure 6:**
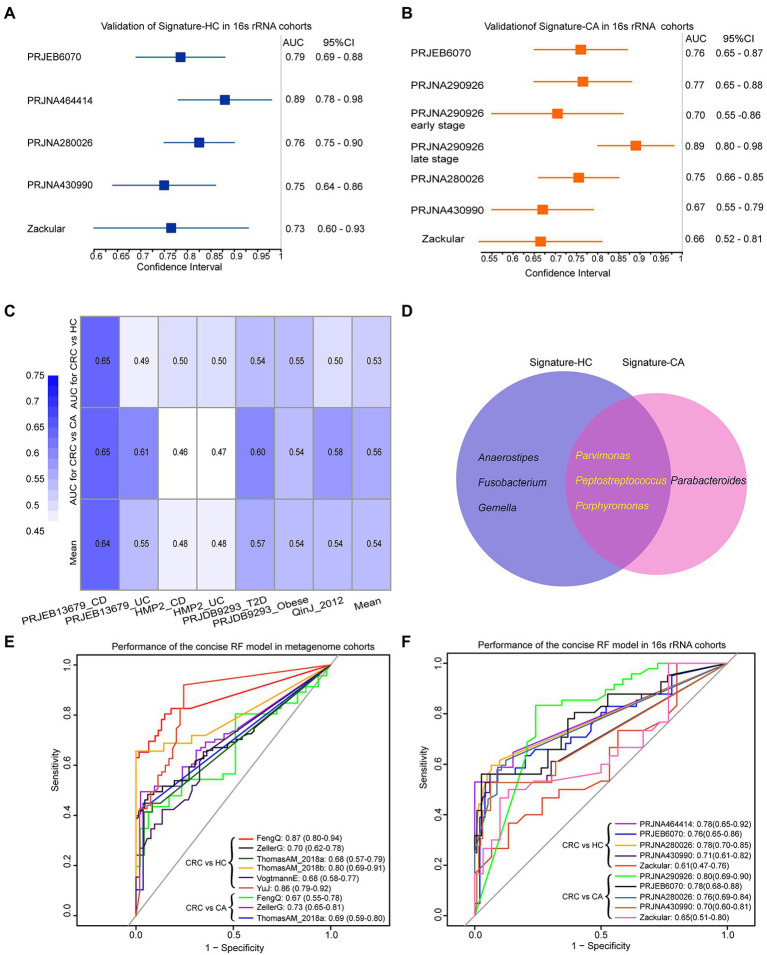
The performances of signature-HC and signature-CA across 16S rRNA gene sequencing data and three common genera shared by signature-HC and signature-CA. Performance of the signature-HC **(A)** and signature-CA **(B)** in six independent 16S rRNA gene sequencing datasets. **(C)** Performance of the signature-HC and signature-CA in other metabolism diseases. **(D)** Venn diagram for signature-HC and signature-CA. Performances of three overlapping genera in six metagenomic cohorts **(E)** and six 16S rRNA gene sequencing cohorts **(F)**.

The two signatures were further validated on six independent 16S rRNA gene sequencing datasets. As shown in Figure 6A, the signature-HC achieved an AUC of 0.79 in PRJEB6070 with 41 CRC and 50 HC samples. The AUC values in Zackular, PRJNA464414, et. ranged from 0.73 to 0.89, with an average of 0.78. Similarly, the signature-CA distinguished CRC from CA in PRJEB6070 with an AUC of 0.76. The AUC values in Zackular, PRJNA290926, et. ranged from 0.66 to 0.77 (Figure 6B). Notably, the signature-CA for distinguishing early-stage and late-stage CRC from CA in PRJNA290926 achieved AUCs of 0.70 (95% CI: 0.55–0.85) and 0.89 (95% CI: 0.80–0.98), respectively. These results demonstrated that the two signatures could be also robustly applied to 16S rRNA gene sequencing data.

### The specificity of microbiological markers for CRC

Four datasets of patients who suffered from CD, UC, T2D and obese were downloaded from curatedMetagenomicData Rpackage and The Inflammatory Bowel Disease Multi’omics Database^4^ to evaluate the specificity of signature-HC and signature-CA (shown in Table 2). The lowest AUC value was 0.46 for CD. As risk factors for CRC, the highest AUC values of CD and UC were 0.65 and 0.61, respectively, which were also lower than that of CRC (Figure 6C). These results pointed out the microbiome markers of CRC that were distinct from that in other metabolism diseases.

**Table 2:** The demographic information of metabolic diseases datasets.

Dataset	Group (Number)	Age (Mean)	BMI (Mean)	Sex (F%/M%)	Technology	Country
PRJEB13679	nonIBD (7)	46.43	NA	42.86/57.14	16S rRNA	United States
	UC (88)	40.14	NA	57.47/42.53		
	CD (167)	35.84	NA	41.57/58.43		
HMP2	nonIBD (429)	NA	NA	45.22/54.78	Shotgun	United States
	UC (459)	23.73	NA	61.22/38.78		
	CD (750)	19.75	NA	63.07/36.93		
PRJDB9293	HC (23)	47.83	NA	0.00/100	16S rRNA	Indonesia
	Obese (27)	45.04	NA	0.00/100		
	T2D (25)	52.08	NA	0.00/100		
QinJ	HC (174)	41.67	23.03	51.73/48.27	Shotgun	China
	T2D (170)	53.52	23.67	37.65/62.35		

### Classification efficiency of three overlapping genera

Notably, three genera, *Porphyromonas*, *Parvimonas* and *Peptostreptococcus*, were overlapped in the two signatures (Figure 6D). As shown in Figure 3D and Supplementary Figure S5, the abundances of these three genera were very low in HC and CA, but were with sharp increases in CRC, indicating their crucial roles in the development of CRC. A concise RF model was built on these three common intestinal florae with optimal parameter combination for mtry = 1 and ntree = 600. In the training dataset FengQ, the concise RF model achieved an AUC of 0.87 for distinguishing CRC from HC samples (95% CI: 0.80–0.94, accuracy: 0.82, sensitivity: 0.65, specificity: 0.95 and F-score:0.77, Figure 6E). The achieved AUC value for distinguishing CRC from HC group in the other five metagenomic cohorts ranged from 0.68 to 0.86. Furthermore, the achieved AUC value for distinguishing CRC from CA group in three metagenomic cohorts ranged from 0.67 to 0.73, with an average of 0.70. In the six 16S rRNA gene sequencing datasets, the AUC of the concise RF model for distinguishing CRC from HC or CA samples ranged from 0.61 to 0.80 (Figure 6F). To sum up, a stool-based diagnostic test using a limited number of intestinal florae would serve as a promising clinical tool.

### Functional gene family associated with bacterial markers

**Figure 7:**
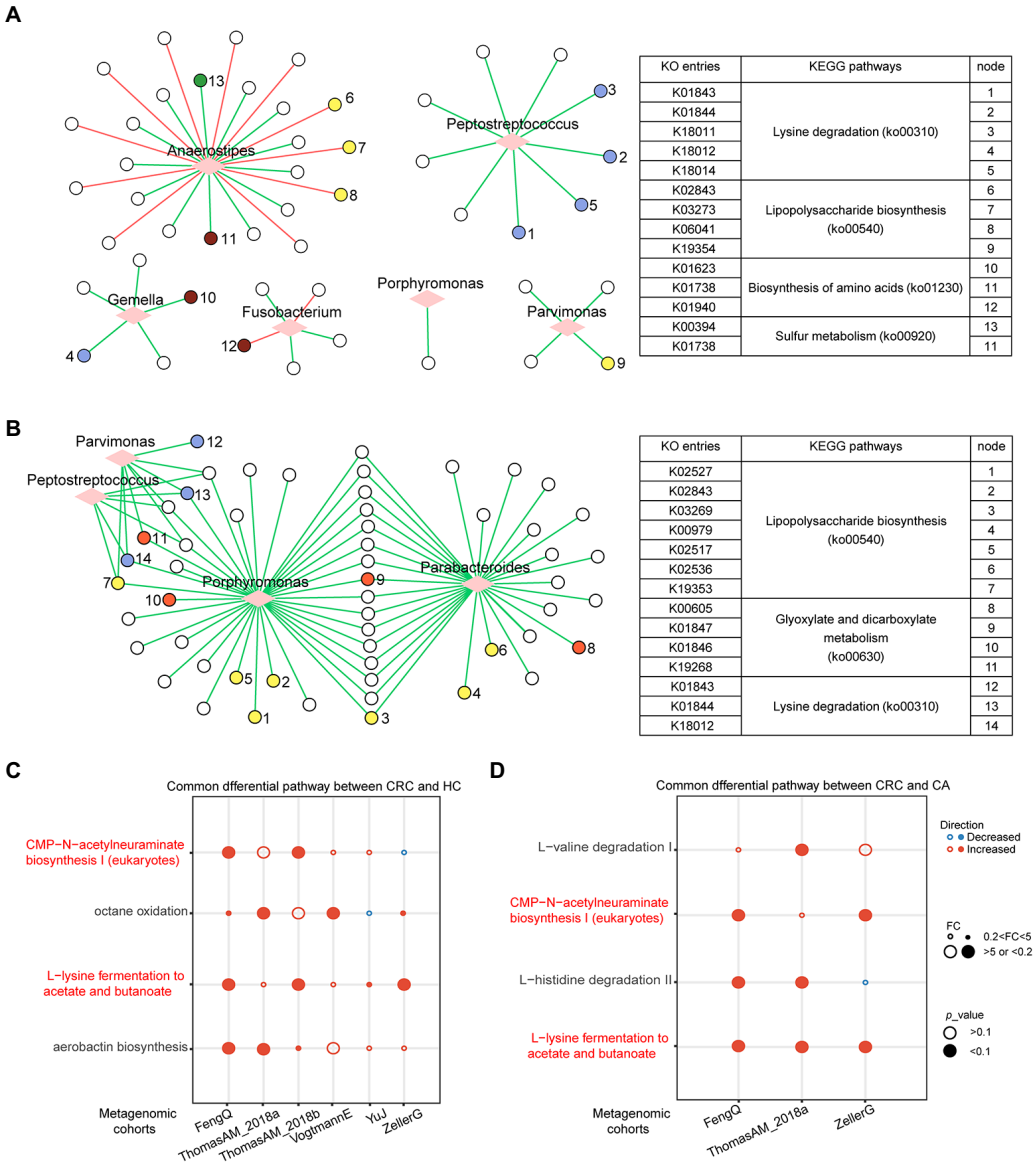
Functional gene family and pathways analysis in metagenomic sequencing datasets. **(A)** Correlation network between six bacterial markers and differential KO categories between CRC and HC. **(B)** Correlation network between four bacterial markers and differential KO categories between CRC and CA. The correlation was evaluated by Spearman. Circle represented KO categories and diamond represented bacterial markers. Red or green edges represented negative or positive correlation. Nodes with the same color share the same KEGG pathway. Consistently dysregulated pathways between CRC and HC **(C)** or CA **(D)**. The overlapped pathways were marked in red.

A total of 4,238 KO categories were identified in six metagenomic cohorts. Using Wilcoxon rank-sum test, 741, 627, 232, 152, 415, and 777 KO categories with distinguishable differential abundance were identified between CRC and HC groups in FengQ, ZellerG, YuJ, ThomasAM_2018b, VogtmannE and ThomasAM_2018a, respectively (*p* < 0.01). Among them, 102 KO categories were enriched in CRC group in at least three cohorts, while 24 KO categories were depleted in CRC group. These KO categories were denoted as CRC-associated KO categories. Spearman correlation analysis showed that 48 CRC-associated KO categories that were significantly associated with the six genera in signature-HC (*p* < 0.05), which were mapped to the KEGG pathways related to cancer occurrence and development, such as ko00310 (Lysine degradation), ko00540 (Lipopolysaccharide biosynthesis; Song et al., 2018), ko00920 (Sulfur metabolism; Ward and DeNicola, 2019) etc. (Figure 7A). Comparing to the CA group, 441, 275, and 43 KO categories were with significantly differential abundance in CRC groups of FengQ, ZellerG and ThomasAM_2018a, respectively (*p* < 0.01), of which 56 KO categories were enriched in the CRC group in at least two cohorts. Spearman correlation analysis showed that 55 KO categories were significantly associated with four bacterial markers in signature-CA, and 17 KEGG pathways with at least two KO categories were mapped, also including ko00310 (Lysine degradation) and ko00540 (Lipopolysaccharide biosynthesis), etc. (Figure 7B). These results indicated that bacterial markers in signature-HC and signature-CA might play important roles in functional alterations of CRC.

### Microbial functional changes in CRC

The microbiome-based functional alterations on different status were examined. Using Fold-Change (FC > 5 or FC < 0.2) and Wilcoxon rank-sum test (*p* < 0.1), 10, 26, 4, 8, 2, and 13 pathways, which had significant differences between CRC and HC groups, were identified in FengQ, ThomasAM_2018a, ZellerG, ThomasAM_2018b, YuJ and VogtmannE, respectively. A total of four pathways were consistently dysregulated in CRC group in at least two datasets (Figure 7C). Similarly, 18, 9, and 7 pathways, which had significant difference between CRC and CA groups, were identified in ThomasAM_2018a, FengQ and ZellerG, respectively. Comparing with CA group, four pathways had consistent dysregulated direction in CRC group in at least two datasets (Figure 7D). In the two sets of dysregulated pathways, CMP-N-acetylneuraminate biosynthesis I (eukaryotes) and L-lysine fermentation to acetate and butanoate were significantly increased in CRC group compared to both HC and CA groups.

## Discussion

This study comprehensively assessed the alterations of gut microbiome from HC, CA to CRC across six metagenomic cohorts from different regions, and identified six and four universal genera with significantly different abundance between CRC and HC or CA at the genus level. Based on these universal genera, two RF models were constructed to distinguish CRC from HC or CA and validated in five metagenomic cohorts and six 16S rRNA gene sequencing datasets. The two RF models were also specific to CRC against metabolic diseases. Moreover, a concise RF model on three common intestinal genera (*Porphyromonas*, *Parvimonas* and *Peptostreptococcus*) distinguished CRC from HC or CA with AUC of 0.87 and 0.67, respectively, which would serve as a promising clinical tool for non-invasive diagnosis of CRC. Functional gene family analysis showed that CRC-associated KO categories were significantly related to bacterial markers in signature-HC and signature-CA and mapped to malignancy-associated pathways.

It has long been reported that fecal bacteria could serve as biomarkers for non-invasive diagnosis of CRC, such as *F. nucleatum*, *Escherichia coli*, and *Bacteroides fragilis* (Yu et al., 2017; Abdelrahman et al., 2019; Shen et al., 2021). However, previous and this work revealed that large variations of microbes existed among studies, indicating the necessity of cross-studies analysis. Metagenomic sequencing and 16S rRNA gene sequencing are the two most useful techniques for studying gut microbiota. For metagenomic sequencing, the coverage of taxonomy is highly dependent on reference genomes and possibly miss some species without known genomes or marker genes, which thus produce biases in relative abundance estimation. In 16S rRNA gene sequencing, microbiota usually annotated to the genus level and the abundance of microbiota is affected by sample concentration, PCR cycle number, amplification primers, etc. However, few microbial markers can be applied to both techniques. As far as we know, this is the first study to explore diagnostic biomarker for CRC in the metagenomic sequencing studies and 16S rRNA gene sequencing studies. The universal microbial biomarkers across populations and technologies in this study would be with great potential application in varieties of clinical scenarios.

Notably, three common intestinal genera (*Porphyromonas*, *Parvimonas* and *Peptostreptococcus*) in the signature-HC and signature-CA, were sharply increased from HC and CA to CRC at the genus level, which were all previously reported as oral pathogens. Increased *Porphyromonas* has been reported in CRC patients from different populations (Wang et al., 2012; Ahn et al., 2013). *Parvimonas* and *Peptostreptococcus* have been also identified to be associated with CRC (Flemer et al., 2017; Yu et al., 2017). In addition, another oral pathogen, *Fusobacterium*, which was sharply increased in CRC samples in FengQ cohort, has been found to be enriched in stools samples from patients with CA and CRC (Marchesi et al., 2011; Castellarin et al., 2012; Kostic et al., 2013). These results suggested that an oral–gut translocation route was associated with CRC, which need further studies to confirm the relationship and elucidate the possible mechanism of these genera in CRC.

Functional gene family analysis revealed that bacterial markers in signature-HC and signature-CA were significantly correlated with most of the CRC-associated KO categories, suggesting that the changes of bacterial markers in CRC may lead to alterations in some functional gene family. Notably, *Anaerostipes* was negatively correlated with many KO categories in CRC, while other six bacterial markers in signature-HC and signature-CA were positively related with the CRC-associated KO categories. Moreover, we also found that the above KO categories were mapped into some malignancy-associated pathways, such as pathways related to lipopolysaccharide (LPS), sulfur metabolism. LPS is a gram-negative bacterial antigen that activates TLR4 and induces immunosuppressive factors and apoptosis resistance, thereby promoting immune escape in human CRC cells (Li et al., 2014). Wu et al. have found that LPS accelerates glycolysis through the nuclear factor-κB/snail/hexokinase3 signaling axis to promote CRC metastasis (Wu et al., 2021a), and Liu et al. have found that activation of p38 mitogen-activated protein kinase pathway by LPS aggravates postoperative intestinal obstruction in CRC patients (Liu et al., 2022). Collectively, LPS might play an important role in the progression of CRC. A variety of molecular species constituted with sulfur atoms are essential to oxidation/reduction (redox) reactions to generate the energy and biomass for tumor growth (Ward and DeNicola, 2019).

The functional analysis showed that L-lysine fermentation to acetate and butanoate was significantly increased in CRC compared to HC and CA groups. Previous studies have found that butyrate, as the preferred energy source for colon cells, maintains mucosal integrity, reduces pro-inflammatory cytokines, and induces apoptosis in colorectal cancer cell lines (Klampfer et al., 2003; Inagaki and Sakata, 2005; Bordonaro et al., 2008; Lazarova et al., 2014). Gao et al. found that CRC patients was characterized by a reduction of butyrate-producing bacteria (Gao et al., 2021) and Weir et al. found that butyric acid was significantly higher in the feces of healthy individuals (Weir et al., 2013). However, conflict results are observed in recent studies. For example, Thomas et al. have found that the CRC-enriched microbiome is positively associated with metabolic pathways that convert different amino acids into L-lysine fermentation to acetate and butyrate pathway (Zeller et al., 2014; Thomas et al., 2019). In this study, L-lysine fermentation to acetate and butanoate was also found to be enriched in CRC. Nevertheless, other pathways for butanoate-producing, which jointly influenced the total amount of butanoate, such as pyruvate fermentation to butanoate and 2-methylbutanoate biosynthesis, were reduced in CRC (Supplementary Table S9). Moreover, the alteration in relative abundance of pyruvate fermentation to butanoate and 2-methylbutanoate biosynthesis was higher than that in L-lysine fermentation to acetate and butanoate. Hence, the alteration of total amount of butanoate in CRC and the vital mechanism of butanoate synthesis needs to be further investigated.

Being a bioinformatics paper, there were some weaknesses in this study. First, animal or cell experiments are needed to confirm the impact of specific gut microbiota and pathways associated with CRC. Second, although we identified the significantly different intestinal flora from hundreds of samples across multiple metagenomic and 16S rRNA gene sequencing datasets, prospective study with more samples is needed to validate the diagnostic values of the two signatures for CRC. Third, in order to obtain the universal microbial markers which can be applied to both 16S rRNA gene sequencing and metagenomic sequencing data, we only constructed universal diagnostic markers at the genus level. More detailed taxonomic model at the species or functional gene family level is needed.

## Conclusion

Two RF models based on six and four universal microbial markers were effective and robust to discriminate CRC from HC or CA in cross-cohorts, regardless of geographic and technical variance, which could serve as an effective clinical indicator for diagnosis of CRC.

## Data availability statement

The 16S rRNA sequencing data can be found in online database. The names of the database and accession number(s) can be found below: SRA - PRJNA464414, PRJEB6070, PRJNA280026,PRJNA290926, PRJNA430990, Microbiome Biomarker CRC - Zackular. The metagenomic sequencing data can be found in curatedMetagenomicData Rpackage.

## Author contributions

Study conception and design were performed by HZ, JW, and LA. Material preparation, public data collection, and analysis were performed by HZ. The first draft of the manuscript was written by HZ. Supervision and revision was done by LA and TC. All authors contributed to the article and approved the submitted version.

## Funding

This work was supported by the Joint research program of health and education in Fujian Province (2019-WJ-32), the Natural Science Foundation of Fujian Province (2020J01600), the open project for Fujian Key Laboratory of Medical Bioinformatics (FKLMB-202001), the National Natural Science Foundation of China (no. 62102065), Joint Funds for innovation of science and Technology, Fujian province (2022J05055) and Fujian Medical University Research Foundation of Talented Scholars (XRCZX2022003).

## Conflict of interest

The authors declare that the research was conducted in the absence of any commercial or financial relationships that could be construed as a potential conflict of interest.

## Publisher’s note

All claims expressed in this article are solely those of the authors and do not necessarily represent those of their affiliated organizations, or those of the publisher, the editors and the reviewers. Any product that may be evaluated in this article, or claim that may be made by its manufacturer, is not guaranteed or endorsed by the publisher.

## Footnotes

^1^https://www.ncbi.nlm.nih.gov/sra (Accessed on December 11, 2021).

^2^http://www.mothur.org/MicrobiomeBiomarkerCRC (Accessed on April 19, 2022).

^3^Release132, http://www.arb-silva.de.

^4^https://ibdmdb.org/tunnel/public/summary.html (Accessed on August 10, 2021).

## Supplementary material

SUPPLEMENTARY FIGURE S1 The flow chart of this study.

SUPPLEMENTARY FIGURE S2 Boxplots of α-diversity in six metagenomic sequencing datasets. **(A)** Shannon index of α-diversity; **(B)** Simpson index; All boxplots represent the 25th–75th percentile of the distribution; the median is shown in a thick line at the middle of the box. Green, HC samples; Pink, CA patients; Blue, CRC patients; ***: *p* < 0.001, **: *p* < 0.01, *: *p* < 0.05, ns: *p* > 0.05.

SUPPLEMENTARY FIGURE S3 PCoA of samples based on Bray–Curtis distance in six metagenomic sequencing datasets. *p*-values of β-diversity based on Bray–Curtis distance were calculated with PERMANOVA.

SUPPLEMENTARY FIGURE S4 Dispersion of samples based on Bray–Curtis distance in six metagenomic sequencing datasets. **(A)** FengQ cohort. **(B)** ThomasAM_2018a cohort. **(C)** ThomasAM_2018b cohort. **(D)** VogtmannE cohort. (E) ZellerG cohort. (F) YuJ cohort.

SUPPLEMENTARY FIGURE S5 Genera that abundance were significantly altered with disease progressed. **(A)** ZellerG cohort. **(B)** ThomasAM_2018a cohort.

SUPPLEMENTARY FIGURE S6 The performances of signature-HC and signature-CA compared to other publications. **(A)** The average AUC of signature-HC resulting from study-to-study transfer validation and the AUC values of the published models in six metagenomic datasets. **(B,C)** The boxplots of AUC values for study-to-study transfer validation of signature-HC (signature-CA) and RF models proposed by Thomas et al. All boxplots represent the 25th–75th percentile of the distribution; the median is shown in a thick line at the middle of the box.
