# Supplementary figures and images for: Microbiome analysis reveals universal diagnostic biomarkers for colorectal cancer across populations and technologies
Source: Front Microbiol. 2022 Nov 3;13:1005201. doi: 10.3389/fmicb.2022.1005201 (PMC9668862; doi:10.3389/fmicb.2022.1005201)

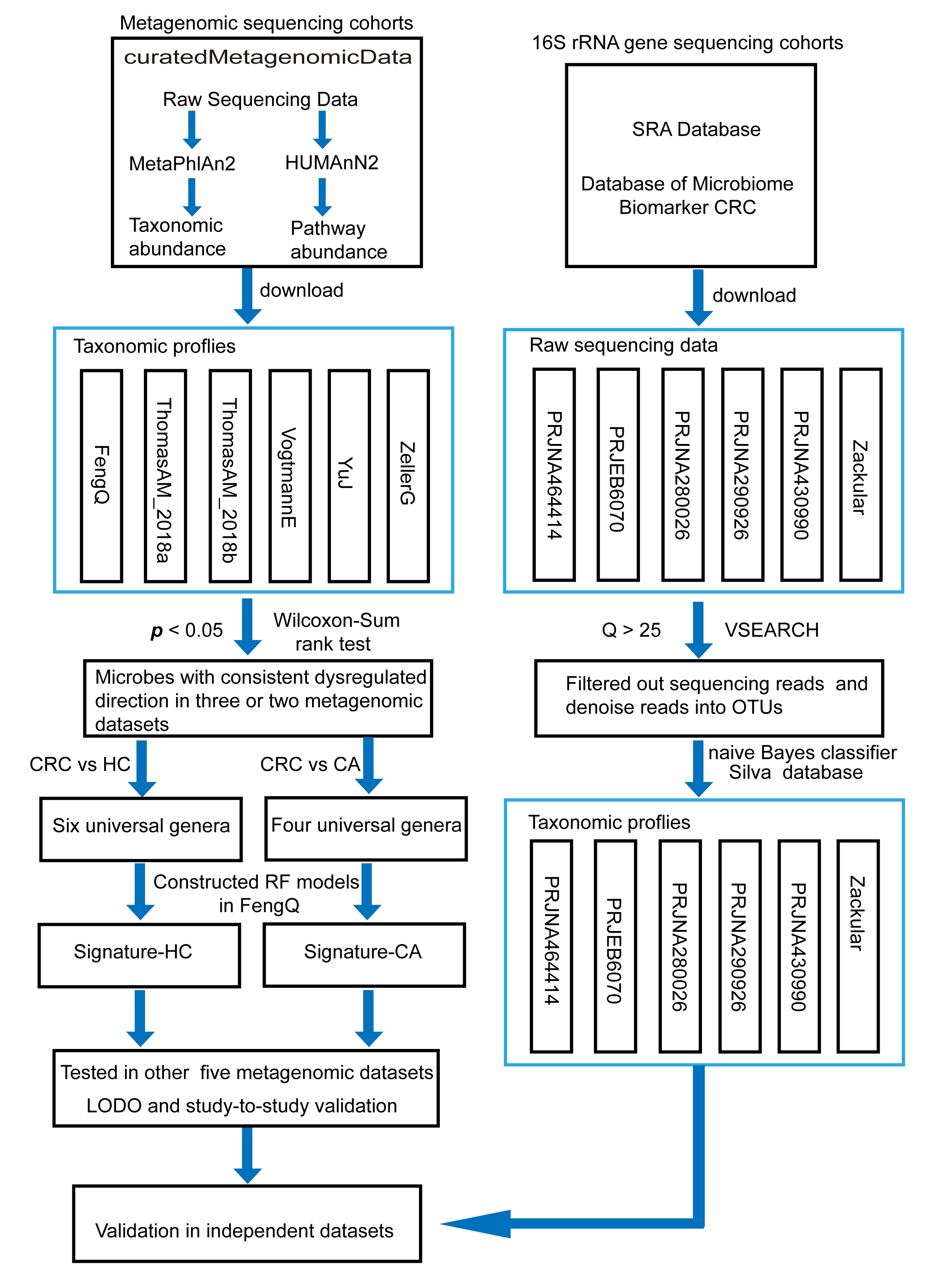

Supplement: SUPPLEMENTARY FIGURE S1 — The flow chart of this study. [file Image_1.TIF]

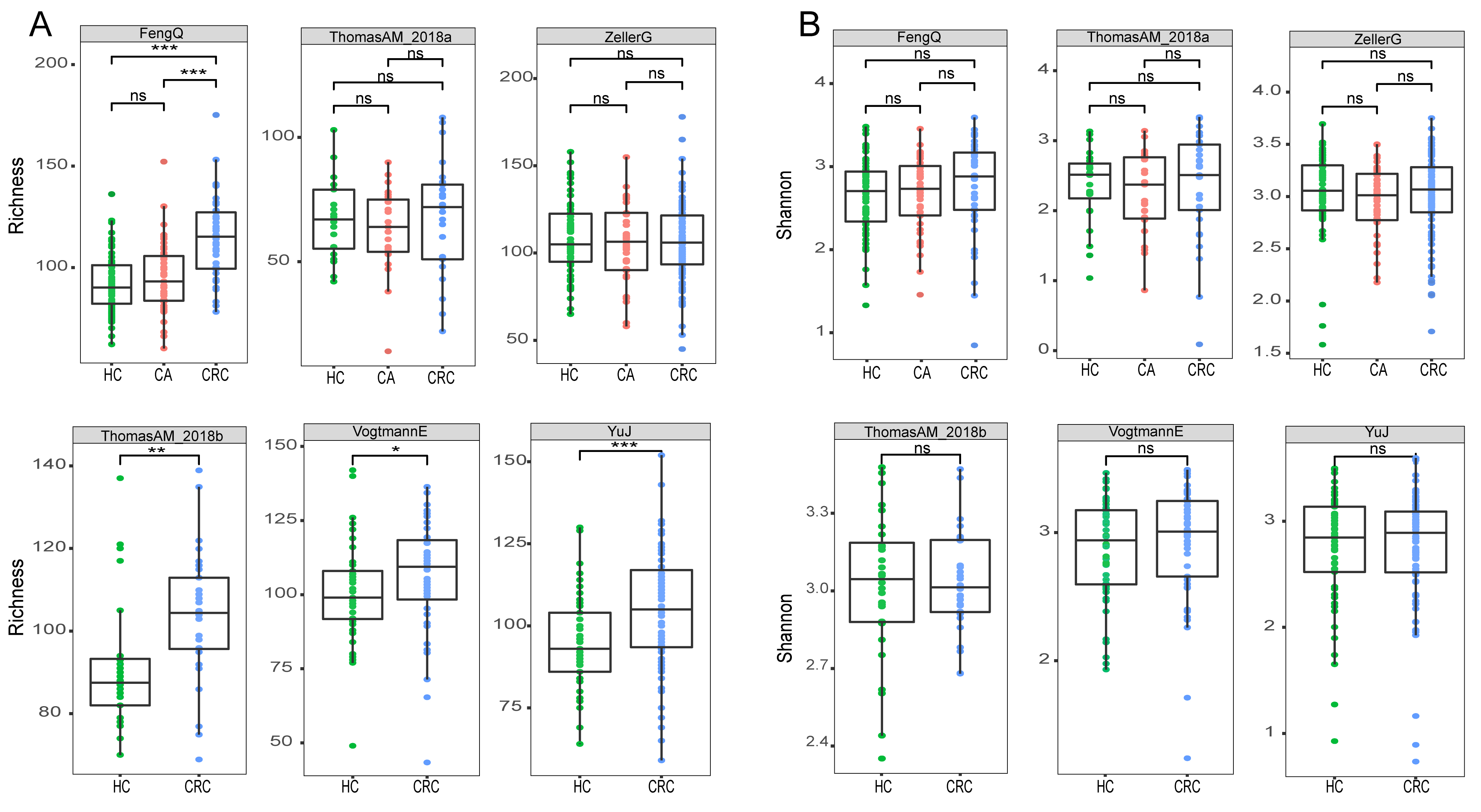

Supplement: SUPPLEMENTARY FIGURE S2 — Boxplots of α-diversity in six metagenomic sequencing datasets. (A) Shannon index of α-diversity; (B) Simpson index; All boxplots represent the 25th–75th percentile of the distribution; the median is shown in a thick line at the middle of the box. Green, HC samples; Pink, CA patients; Blue, CRC patients; ***: p < 0.001, **: p < 0.01, *: p < 0.05, ns: p > 0.05. [file Image_2.tif]

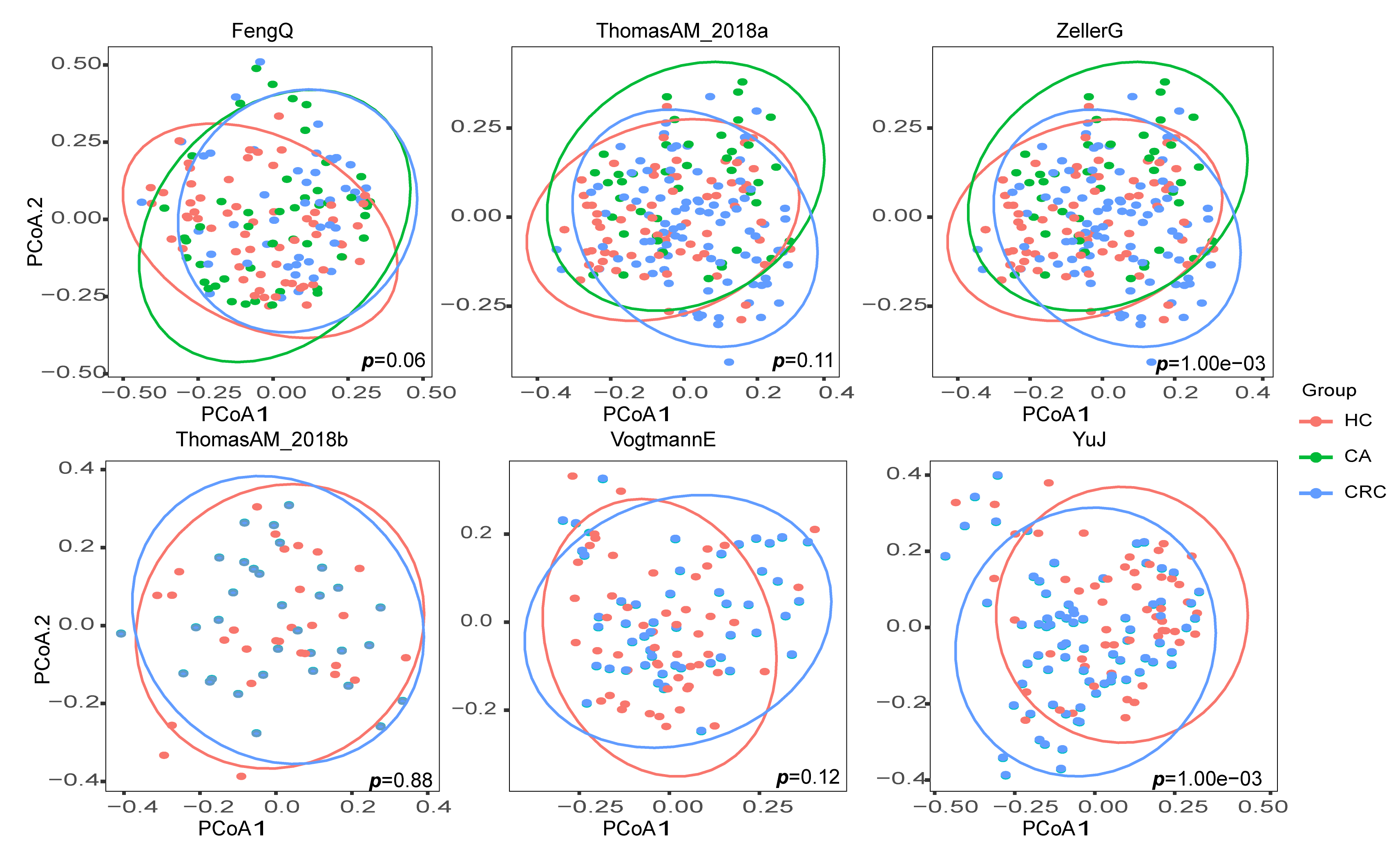

Supplement: SUPPLEMENTARY FIGURE S3 — PCoA of samples based on Bray–Curtis distance in six metagenomic sequencing datasets. p-values of β-diversity based on Bray–Curtis distance were calculated with PERMANOVA. [file Image_3.TIF]

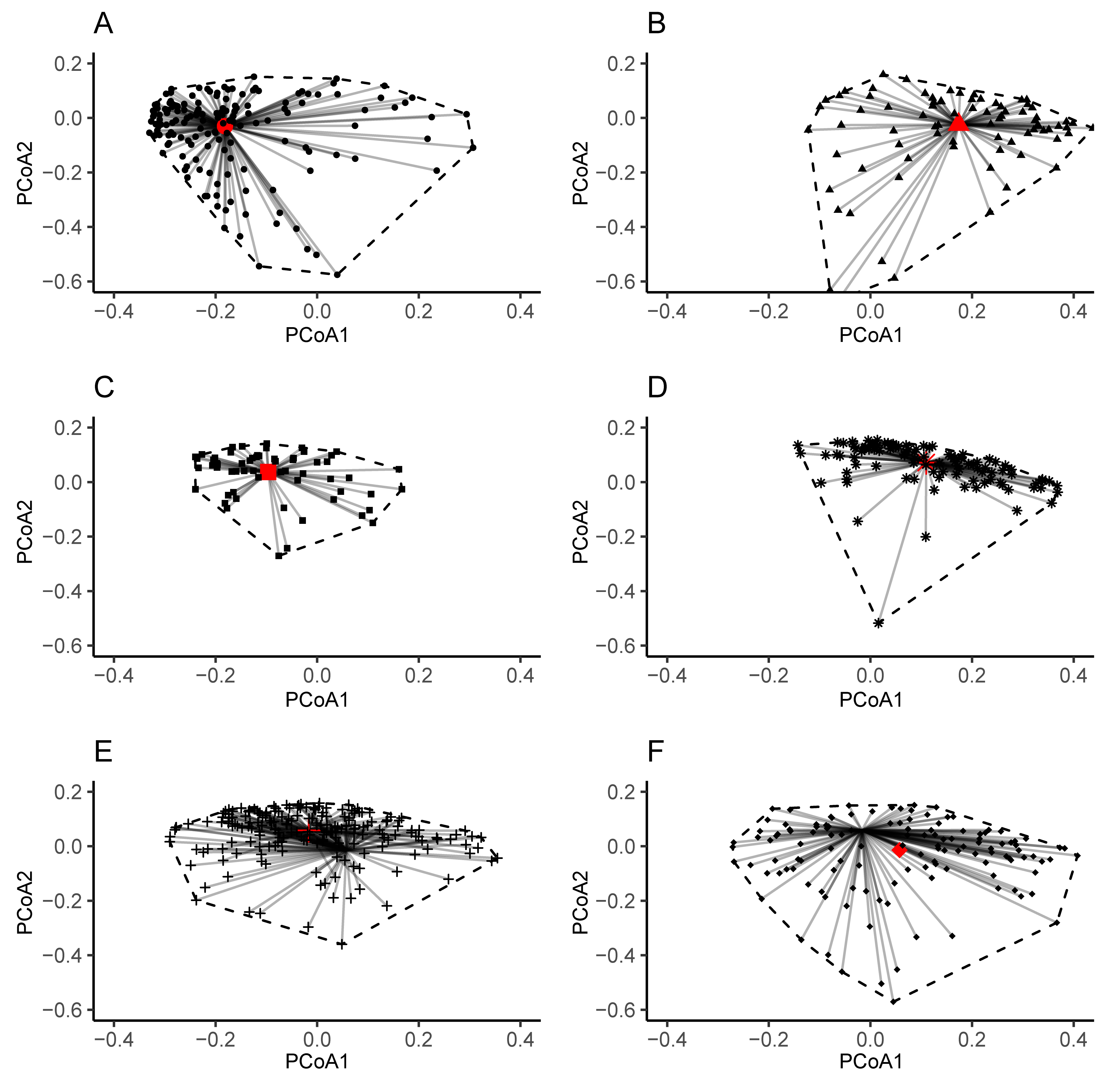

Supplement: SUPPLEMENTARY FIGURE S4 — Dispersion of samples based on Bray–Curtis distance in six metagenomic sequencing datasets. (A) FengQ cohort. (B) ThomasAM_2018a cohort. (C) ThomasAM_2018b cohort. (D) VogtmannE cohort. (E) ZellerG cohort. (F) YuJ cohort. [file Image_4.tif]

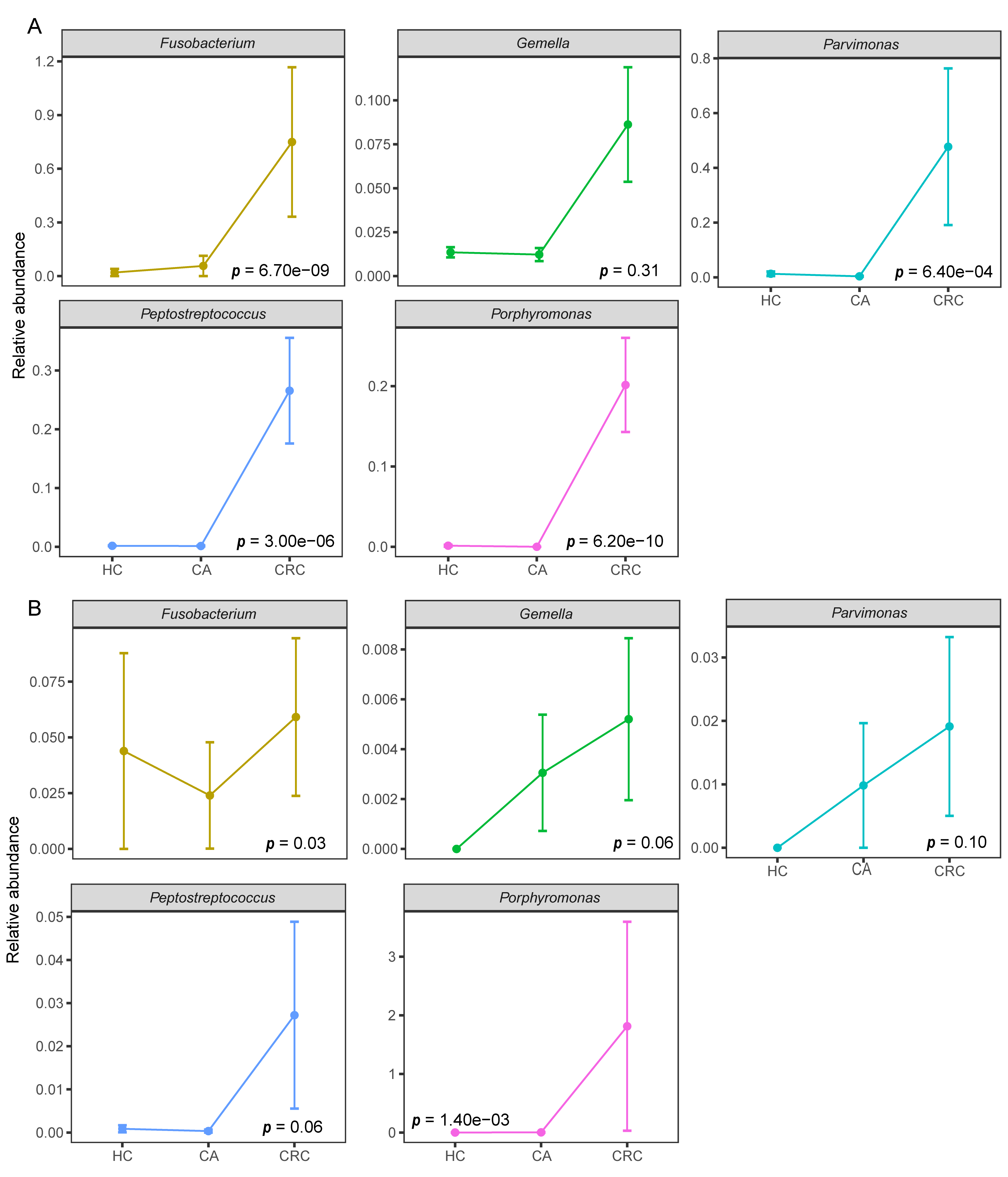

Supplement: SUPPLEMENTARY FIGURE S5 — Genera that abundance were significantly altered with disease progressed. (A) ZellerG cohort. (B) ThomasAM_2018a cohort. [file Image_5.TIF]

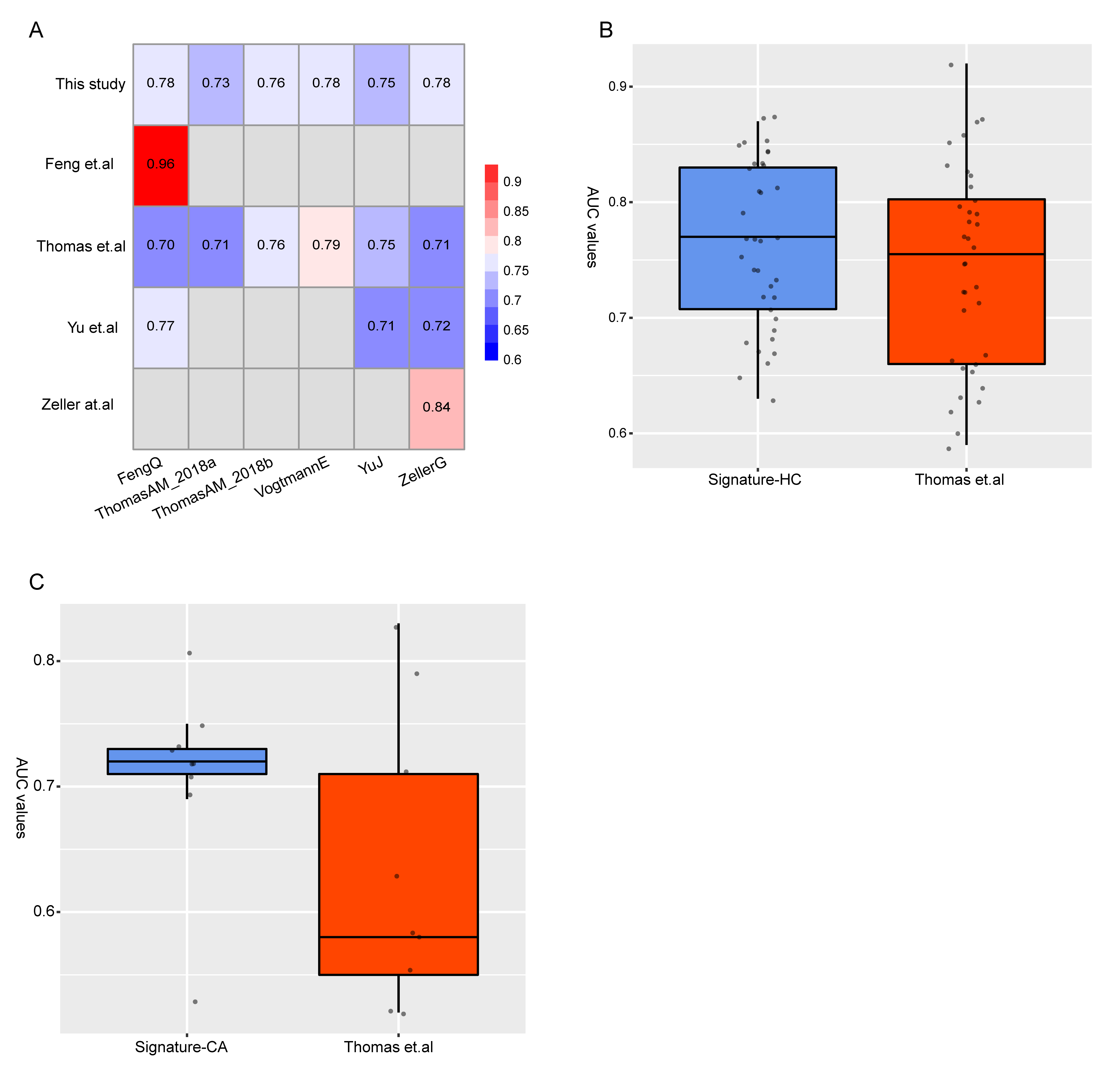

Supplement: SUPPLEMENTARY FIGURE S6 — The performances of signature-HC and signature-CA compared to other publications. (A) The average AUC of signature-HC resulting from study-to-study transfer validation and the AUC values of the published models in six metagenomic datasets. (B,C) The boxplots of AUC values for study-to-study transfer validation of signature-HC (signature-CA) and RF models proposed by Thomas et al. All boxplots represent the 25th–75th percentile of the distribution; the median is shown in a thick line at the middle of the box. [file Image_6.TIF]
