# Supplementary material for: Microbiome analysis reveals universal diagnostic biomarkers for colorectal cancer across populations and technologies
Source: Front Microbiol. 2022 Nov 3;13:1005201. doi: 10.3389/fmicb.2022.1005201 (PMC9668862; doi:10.3389/fmicb.2022.1005201)
Supplement: Supplementary file 7 [file Table_1.docx]

**Supplementary Tables**

**Table S1. Differential phylum between CRC and HC in six metagenomic datasets.**

| **Phylum** | **FengQ** | | **ThomasAM_2018a** | | **ThomasAM_2018b** | | **VogtmannE** | | **YuJ** | | **ZellerG** | |
| --- | --- | --- | --- | --- | --- | --- | --- | --- | --- | --- | --- | --- |
|  | ***P-value*** | **Dir** | ***P-value*** | **Dir** | ***P-value*** | **Dir** | ***P-value*** | **Dir** | ***P-value*** | **Dir** | ***P-value*** | **Dir** |
| **Firmicutes** | 8.30e-03 | -1 | 6.43e-03 | -1 | - | - | - | - | 4.57e-02 | -1 | 5.58e-04 | -1 |
| **Bacteroidetes** | 2.35e-04 | 1 | - | - | - | - | - | - | - | - | 2.73e-04 | 1 |
| **Actinobacteria** | 4.96e-03 | -1 | - | - | - | - | - | - | - | - | 2.90e-03 | -1 |
| **Proteobacteria** | 5.36e-03 | 1 | 3.78e-03 | 1 | - | - | 4.56e-02 | 1 | - | - | - | - |
| **Verrucomicrobia** | - | - | - | - | - | - | - | - | - | - | - | - |
| **Fusobacteria** | 1.14e-09 | 1 | 3.54e-02 | 1 | 1.65e-03 | 1 | 8.10e-04 | 1 | 7.28e-07 | 1 | 2.04e-07 | 1 |
| **Euryarchaeota** | 2.80e-03 | 1 | - | - | 1.55e-03 | 1 | - | - | - | - | - | - |
| **Synergistetes** | - | - | - | - | - | - | 1.73e-02 | 1 | - | - | - | - |

Note: 1: Upregulated; -1: Downregulated.

**Table S2. Differential phylum between CRC and CA in three metagenomic datasets.**

| **Phylum** | **FengQ** | | **ThomasAM_2018a** | | **ZellerG** | |
| --- | --- | --- | --- | --- | --- | --- |
|  | ***P-value*** | **Dir** | ***P-value*** | **Dir** | ***P-value*** | **Dir** |
| **Firmicutes** | 1.16e-02 | -1 | - | - | 8.64e-03 | -1 |
| **Bacteroidetes** | 7.52e-03 | 1 | - | - | 4.86e-02 | 1 |
| **Actinobacteria** | - | - | 4.14e-02 | -1 | - | - |
| **Proteobacteria** | 1.27e-02 | 1 | 0.41 | 1 | - | - |
| **Verrucomicrobia** | - | - | - | - | - | - |
| **Fusobacteria** | 1.96e-04 | -1 | - | - | 8.96e-06 | 1 |
| **Euryarchaeota** | - | - | - | - | - | - |
| **Synergistetes** | - | - | - | - | - | - |

Note: 1: Upregulated; -1: Downregulated.

**Table S3. Differential genera between CRC and HC in six metagenomic datasets.**

| **Genus** | **ThomasAM_2018a** | | **ThomasAM_2018b** | | **VogtmannE** | | **YuJ** | | **ZellerG** | | **FengQ** | | |
| --- | --- | --- | --- | --- | --- | --- | --- | --- | --- | --- | --- | --- | --- |
|  | ***P-value*** | **Dir** | ***P-value*** | **Dir** | ***P-value*** | **Dir** | ***P-value*** | **Dir** | ***P-value*** | **Dir** | ***P-value*** | **Dir** |  |
| **Abiotrophia** | 1.12e-02 | 1 | - | - | - | - | - | - | 2.55e-02 | -1 | - | - |  |
| **Acidaminococcaceae_unclassified** | - | - | - | - | - | - | - | - | 1.28e-02 | 1 | - | - |  |
| **Acidaminococcus** | - | - | - | - | - | - | - | - | - | - | 8.87e-03 | 1 |  |
| **Actinomyces** | - | - | 1.81e-02 | 1 | - | - | 3.27e-02 | 1 | - | - | - | - |  |
| **Adlercreutzia** | - | - | - | - | 2.53e-02 | -1 | - | - | - | - | - | - |  |
| **Alistipes** | - | - | - | - | - | - | - | - | 4.14e-02 | 1 | 2.25e-03 | 1 |  |
| **Alloscardovia** | - | - | - | - | 4.40e-02 | -1 | - | - | - | - | - | - |  |
| **Anaerococcus** | - | - | 3.17e-02 | 1 | 5.86e-03 | 1 | - | - | 2.57e-02 | 1 | 6.25e-04 | 1 |  |
| **Anaerostipes** | 3.10e-02 | -1 | - | - | - | - | - | - | 1.84e-03 | -1 | 2.39e-03 | -1 |  |
| **Anaerotruncus** | - | - | - | - | - | - | 4.64e-02 | 1 | 4.54e-02 | 1 | 2.63e-03 | 1 |  |
| **Atopobium** | - | - | 4.23e-04 | 1 | - | - | 2.41e-03 | 1 | - | - | - | - |  |
| **Bacteroidales**  **_noname** | - | - | - | - | 1.02e-02 | -1 | - | - | - | - | 2.15e-02 | 1 |  |
| **Bacteroides** | - | - | - | - | - | - | - | - | 3.77e-02 | 1 | 6.02e-03 | 1 |  |
| **Barnesiella** | - | - | - | - | - | - | 1.61e-02 | 1 | - | - | 1.32e-02 | 1 |  |
| **Bifidobacterium** | - | - | - | - | - | - | - | - | 2.52e-03 | -1 | 2.18e-02 | -1 |  |
| **Bilophila** | - | - | - | - | - | - | - | - | 1.10e-03 | 1 | 1.96e-02 | 1 |  |
| **Blautia** | - | - | - | - | 2.27e-03 | 1 | 9.86e-03 | 1 | - | - | - | - |  |
| **Burkholderiales**  **_noname** | - | - | - | - | - | - | 3.26e-02 | -1 | - | - | - | - |  |
| **Butyricicoccus** | - | - | - | - | - | - | - | - | - | - | 6.93e-03 | 1 |  |
| **C2likevirus** | - | - | - | - | - | - | - | - | - | - | 1.89e-02 | 1 |  |
| **Campylobacter** | - | - | - | - | - | - | - | - | 4.04e-04 | 1 | 7.76e-03 | 1 |  |
| **Catenibacterium** | - | - | - | - | - | - | 2.28e-02 | -1 | - | - | 2.12e-02 | 1 |  |
| **Citrobacter** | - | - | - | - | 2.54e-02 | -1 | - | - | - | - | 1.29e-02 | 1 |  |
| **Clostridiaceae**  **_noname** | - | - | - | - | - | - | - | - | - | - | 3.68e-02 | -1 |  |
| **Clostridiales_Family_XIII_Incertae_Sedis_noname** | - | - | - | - | - | - | 4.38e-03 | 1 | - | - | - | - |  |
| **Clostridiales_Family_XIII_Incertae_Sedis_unclassified** | - | - | 3.20e-02 | 1 | - | - | 3.88e-05 | 1 | - | - | - | - |  |
| **Clostridiale**  **s_noname** | - | - | - | - | - | - | 1.02e-03 | 1 | - | - | - | - |  |
| **Clostridium** | - | - | - | - | - | - | 1.34e-03 | 1 | - | - | 7.01e-03 | 1 |  |
| **Collinsella** | - | - | - | - | - | - | 3.61e-02 | 1 | - | - | - | - |  |
| **Coprobacillus** | - | - | - | - | - | - | 6.59e-04 | 1 | - | - | - | - |  |
| **Coprobacter** | - | - | - | - | - | - | - | - | - | - | 1.23e-02 | 1 |  |
| **Coprococcus** | - | - | - | - | - | - | 2.31 e-03 | -1 | - | - | - | - |  |
| **Desulfovibrio** | - | - | - | - | - | - | - | - | - | - | 2.81e-02 | 1 |  |
| **Dialister** | - | - | - | - | - | - | - | - | - | - | 1.16e-04 | 1 |  |
| **Eggerthella** | - | - | - | - | - | - | 2.79e-02 | 1 | - | - | - | - |  |
| **Eikenella** | 2.02e-02 | 1 | - | - | - | - | 9.39e-03 | 1 | 8.87e-03 | 1 | 2.00e-02 | 1 |  |
| **Enterobacter** | - | - | - | - | 7.55e-03 | -1 | - | - | - | - | - | - |  |
| **Enterococcus** | - | - | - | - | - | - | - | - | 3.96e-03 | -1 | - | - |  |
| **Escherichia** | 5.46e-03 | 1 | 2.28e-02 | 1 | 2.35e-02 | 1 | - | - | - | - | 8.45e-03 | 1 |  |
| **Eubacterium** | 3.11e-02 | -1 | - | - | - | - | 6.32e-05 | -1 | 1.06e-03 | -1 | 4.85e-02 | -1 |  |
| **Faecalibacterium** | 4.43e-02 | -1 | - | - | - | - | 5.83e-04 | -1 | - | - | - | - |  |
| **Flavonifractor** | - | - | - | - | - | - | - | - | 1.09e-03 | 1 | 5.22e-03 | 1 |  |
| **Fusobacterium** | 3.54e-02 | 1 | 1.65e-03 | 1 | 2.22e-03 | 1 | 4.36e-07 | 1 | 6.60e-07 | 1 | 1.14e-09 | 1 |  |
| **Gemella** | 2.02e-02 | 1 | 1.61e-02 | 1 | - | - | 1.97e-03 | 1 | - | - | 1.69e-02 | 1 |  |
| **Gordonibacter** | 4.39e-03 | -1 | - | - | - | - | - | - | - | - | - | - |  |
| **Haemophilus** | - | - | - | - | - | - | 1.15e-03 | -1 | - | - | 4.90e-03 | 1 |  |
| **Klebsiella** | - | - | - | - | - | - | - | - | - | - | 3.09e-03 | 1 |  |
| **Lachnospiraceae**  **_noname** | - | - | 2.13e-02 | -1 | - | - | - | - | - | - | - | - |  |
| **Lactobacillus** | - | - | - | - | - | - | - | - | 1.15e-03 | -1 | - | - |  |
| **Leptotrichia** | - | - | - | - | - | - | - | - | 2.06e-02 | 1 | - | - |  |
| **Leptotrichiaceae**  **_unclassified** | - | - | - | - | - | - | - | - | 3.25e-02 | 1 | 4.52e-02 | 1 |  |
| **Megasphaera** | - | - | - | - | - | - | - | - | - | - | 2.17e-03 | 1 |  |
| **Methanobrevibacter** | - | - | 1.72e-03 | 1 | - | - | - | - | - | - | 3.02 e-04 | 1 |  |
| **Morganella** | 2.69e-02 | 1 | - | - | - | - | 3.63e-02 | 1 | 3.45e-02 | 1 | - | - |  |
| **Odoribacter** | - | - | 1.06e-02 | 1 | - | - | - | - | - | - | 2.28e-03 | 1 |  |
| **Olsenella** | - | - | 1.78e-02 | 1 | - | - | - | - | - | - | - | - |  |
| **Oscillibacter** | - | - | - | - | - | - | - | - | 8.62e-04 | 1 | 3.90e-04 | 1 |  |
| **Parabacteroides** | - | - | - | - | - | - | - | - | - | - | 7.11e-03 | 1 |  |
| **Paraprevotella** | - | - | - | - | - | - | - | - | - | - | 2.41e-02 | 1 |  |
| **Parasutterella** | - | - | - | - | - | - | 1.46e-02 | -1 | - | - | - | - |  |
| **Parvimonas** | - | - | 9.50e-06 | 1 | 2.74e-03 | 1 | 8.00e-10 | 1 | 3.97e-03 | 1 | 1.79e-07 | 1 |  |
| **Peptostreptococcaceae_noname** | - | - | - | - | - | - | - | - | 0.008511725 | -1 | - | - |  |
| **Peptostreptococcus** | - | - | 5.04e-05 | 1 | 6.81e-03 | 1 | 1.11e-12 | 1 | 1.32e-04 | 1 | 2.65e-07 | 1 |  |
| **Porphyromonas** | 7.54e-03 | 1 | - | - | 4.20e-03 | 1 | 5.21e-04 | 1 | 2.43e-07 | 1 | 4.76e-09 | 1 |  |
| **Prevotella** | - | - | - | - | - | - | - | - | - | - | 5.85e-11 | 1 |  |
| **Pseudoflavonifractor** | - | - | - | - | - | - | - | - | 2.22e-03 | 1 | 7.15e-04 | 1 |  |
| **Pseudomonas** | - | - | - | - | - | - | - | - | - | - | 3.74e-03 | -1 |  |
| **Roseburia** | - | - | - | - | - | - | 2.67e-02 | -1 | - | - | - | - |  |
| **Rothia** | - | - | - | - | - | - | 4.17e-02 | 1 | - | - | - | - |  |
| **Ruminococcaceae**  **_noname** | - | - | - | - | - | - | - | - | 2.51e-02 | 1 | 1.12e-06 | 1 |  |
| **Ruminococcus** | - | - | - | - | - | - | - | - | 1.45e-04 | -1 | 9.10e-03 | -1 |  |
| **Selenomonas** | - | - | - | - | - | - | - | - | - | - | 2.00e-02 | 1 |  |
| **Solobacterium** | - | - | 4.59e-03 | 1 | 3.98e-02 | 1 | 3.70e-06 | 1 | 1.43e-03 | 1 | 1.26e-02 | 1 |  |
| **Streptococcus** | - | - | - | - | - | - | - | - | 8.11e-03 | -1 | 1.28e-02 | -1 |  |
| **Subdoligranulum** | 3.83e-02 | -1 | - | - | - | - | - | - | 1.90e-02 | 1 | - | - |  |
| **Sutterella** | - | - | - | - | - | - | - | - | - | - | 6.19e-04 | 1 |  |

Note: 1: Upregulated; -1: Downregulated.

**Table S4. Differential genera between CRC and CA in three metagenomic datasets.**

| **Genus** | **ThomasAM_2018a** | | **ZellerG** | | **FengQ** | |
| --- | --- | --- | --- | --- | --- | --- |
|  | ***P-value*** | **Dir** | ***P-value*** | **Dir** | ***P-value*** | **Dir** |
| **Acidaminococcus** | - | - | - | - | 5.42e-05 | 1 |
| **Alistipes** | - | - | - | - | 1.70e-02 | 1 |
| **Alloprevotella** | 4.95e-02 | 1 | - | - | - | - |
| **Anaerococcus** | - | - | - | - | 3.29e-03 | 1 |
| **Anaerotruncus** | - | - | - | - | 2.64e-02 | 1 |
| **Bacteroides** | - | - | 3.38e-02 | 1 | - | - |
| **Barnesiella** | - | - | - | - | 6.65e-04 | 1 |
| **Bilophila** | - | - | - | - | 4.59e-02 | 1 |
| **Burkholderiales_noname** | - | - | - | - | 5.97e-03 | 1 |
| **Butyricicoccus** | - | - | - | - | 1.37e-02 | 1 |
| **C2likevirus** | - | - | - | - | 1.82e-02 | 1 |
| **Campylobacter** | - | - | 1.29e-02 | 1 | - | - |
| **Catenibacterium** | - | - | - | - | 3.46e-02 | 1 |
| **Clostridiales_Family_XIII_Incertae_Sedis_noname** | - | - | - | - | 2.94e-02 | -1 |
| **Collinsella** | 2.52e-02 | -1 | - | - | - | - |
| **Coprobacter** | - | - | - | - | 1.35e-02 | 1 |
| **Dorea** | - | - | 3.50e-02 | -1 | - | - |
| **Eikenella** | - | - | 3.61e-02 | 1 | 4.10e-02 | 1 |
| **Enterobacteriaceae_noname** | - | - | 1.83e-02 | -1 | - | - |
| **Escherichia** | - | - | - | - | 6.00e-03 | 1 |
| **Eubacterium** | - | - | 3.83e-03 | -1 | - | - |
| **Fusobacterium** | - | - | 9.46e-06 | 1 | 1.89e-04 | -1 |
| **Gemella** | - | - | - | - | 1.90e-02 | 1 |
| **Haemophilus** | - | - | - | - | 3.85e-02 | 1 |
| **Holdemania** | - | - | - | - | 3.86e-03 | 1 |
| **Klebsiella** | - | - | - | - | 2.71e-03 | 1 |
| **Lachnospiraceae_noname** | - | - | - | - | 2.41e-02 | -1 |
| **Leptotrichia** | - | - | 2.66e-02 | 1 | - | - |
| **Megasphaera** | - | - | - | - | 3.90e-02 | -1 |
| **Morganella** | 3.77e-03 | 1 | - | - | - | - |
| **Odoribacter** | - | - | - | - | 3.51e-02 | 1 |
| **Oscillibacter** | - | - | - | - | 1.19e-02 | 1 |
| **Parabacteroides** | - | - | 2.66e-02 | 1 | 1.04e-03 | 1 |
| **Parasutterella** | - | - | - | - | 9.51e-03 | 1 |
| **Parvimonas** | - | - | 9.50e-04 | 1 | 1.42e-04 | 1 |
| **Peptostreptococcus** | 3.56e-02 | 1 | 2.40e-05 | 1 | 1.99e-04 | 1 |
| **Porphyromonas** | 5.28e-03 | 1 | 5.50e-06 | 1 | 1.26e-03 | 1 |
| **Prevotella** | - | - | - | - | 3.47e-06 | 1 |
| **Proteus** | 4.95e-02 | 1 | - | - | - | - |
| **Pseudoflavonifractor** | - | - | - | - | 1.14e-02 | 1 |
| **Pseudomonas** | 4.28e-02 | 1 | - | - | - | - |
| **Raoultella** | 4.95e-02 | 1 | - | - | - | - |
| **Ruminococcaceae_noname** | - | - | - | - | 7.35e-04 | 1 |
| **Selenomonas** | - | - | - | - | 4.10e-02 | 1 |
| **Siphoviridae_noname** | 4.95e-02 | 1 | - | - | 5.25e-03 | 1 |
| **Solobacterium** | - | - | 2.64e-03 | 1 | - | - |

Note: 1: Upregulated; -1: Downregulated.

**Table S5. Differential species between CRC and HC in six metagenomic datasets.**

| **Species** | **ThomasAM_2018a** | | **ThomasAM_2018b** | | **VogtmannE** | | **YuJ** | | **ZellerG** | | **FengQ** | |
| --- | --- | --- | --- | --- | --- | --- | --- | --- | --- | --- | --- | --- |
|  | ***P-value*** | **Dir** | ***P-value*** | **Dir** | ***P-value*** | **Dir** | ***P-value*** | **Dir** | ***P-value*** | **Dir** | ***P-value*** | **Dir** |
| **Abiotrophia_defectiva** | 1.12e-02 | 1 | - | - | - | - | - | - | 2.55e-02 | -1 | - | - |
| **Acidaminococcus**  **_sp_HPA0509** | - | - | - | - | 4.34e-02 | 1 | - | - | - | - | - | - |
| **Acidaminococcus**  **_unclassified** | - | - | - | - | - | - | 3.43e-02 | 1 | - | - | 1.34e-02 | 1 |
| **Actinomyces**  **_cardiffensis** | - | - | - | - | - | - | 3.63e-02 | 1 | 1.39e-02 | 1 | - | - |
| **Actinomyces**  **_massiliensis** | - | - | - | - | - | - | - | - | 9.35e-03 | -1 | - | - |
| **Actinomyces**  **_naeslundii** | - | - | - | - | - | - | - | - | 4.91e-03 | -1 | 1.09e-02 | -1 |
| **Actinomyces**  **_odontolyticus** | - | - | - | - | - | - | - | - | 2.16e-02 | 1 | - | - |
| **Actinomyces_oris** | - | - | - | - | - | - | - | - | 6.60e-04 | -1 | - | - |
| **Actinomyces_viscosus** | - | - | 3.15e-02 | 1 | - | - | - | - | 4.64e-02 | -1 | - | - |
| **Adlercreutzia**  **_equolifaciens** | - | - | - | - | 2.53e-02 | -1 | - | - | - | - | - | - |
| **Aggregatibacter**  **_unclassified** | - | - | - | - | - | - | 3.93e-03 | 1 | - | - | - | - |
| **Alistipes_finegoldii** | - | - | - | - | - | - | - | - | - | - | 7.40e-05 | 1 |
| **Alistipes_indistinctus** | - | - | - | - | - | - | 3.08e-02 | 1 | - | - | 3.36e-02 | 1 |
| **Alistipes_onderdonkii** | - | - | - | - | - | - | - | - | - | - | 6.16e-04 | 1 |
| **Alistipes_putredinis** | - | - | - | - | - | - | - | - | - | - | 1.22e-02 | 1 |
| **Alistipes_senegalensis** | - | - | 3.27e-02 | 1 | - | - | - | - | - | - | - | - |
| **Alistipes_sp_AP11** | - | - | - | - | - | - | - | - | 2.93e-02 | 1 | 1.37e-02 | 1 |
| **Alistipes_sp_HGB5** | - | - | - | - | 2.51e-02 | 1 | - | - | - | - | - | - |
| **Alloscardovia**  **_omnicolens** | - | - | - | - | 4.40e-02 | -1 | - | - | - | - | - | - |
| **Anaerococcus**  **_lactolyticus** | - | - | - | - | 4.34e-02 | 1 | - | - | - | - | - | - |
| **Anaerococcus**  **_obesiensis** | - | - | - | - | 1.34e-02 | 1 | - | - | 3.18e-02 | 1 | 8.91e-03 | 1 |
| **Anaerococcus**  **_vaginalis** | - | - | - | - | 2.03e-02 | 1 | - | - | 2.65e-02 | 1 | 6.25e-04 | 1 |
| **Anaerofustis**  **_stercorihominis** | - | - | - | - | - | - | 2.31e-02 | 1 | - | - | - | - |
| **Anaerostipes_hadrus** | 3.10e-02 | -1 | - | - | - | - | - | - | 4.63e-04 | -1 | 2.88e-02 | -1 |
| **Anaerotruncus**  **_colihominis** | - | - | - | - | - | - | 1.81e-02 | 1 | - | - | - | - |
| **Anaerotruncus**  **_unclassified** | - | - | 4.75e-03 | 1 | - | - | - | - | - | - | 8.10e-03 | 1 |
| **Atopobium**  **_parvulum** | - | - | - | - | - | - | 2.77e-03 | 1 | - | - | - | - |
| **Atopobium_rimae** | - | - | - | - | - | - | 2.39e-03 | 1 | - | - | - | - |
| **Avian_endogenous**  **_retrovirus_EAV_HP** | - | - | - | - | - | - | 3.87e-02 | -1 | - | - | - | - |
| **Bacteroidales**  **_bacterium_ph8** | - | - | - | - | 1.02e-02 | -1 | - | - | - | - | 2.15e-02 | 1 |
| **Bacteroides_caccae** | - | - | - | - | - | - | 1.86e-02 | 1 | - | - | 8.38e-03 | 1 |
| **Bacteroides_clarus** | - | - | - | - | - | - | 1.51e-02 | -1 | - | - | - | - |
| **Bacteroides**  **_coprophilus** | - | - | - | - | - | - | - | - | - | - | 1.48e-04 | 1 |
| **Bacteroides_dorei** | - | - | - | - | - | - | - | - | - | - | 1.27e-02 | 1 |
| **Bacteroides_eggerthii** | - | - | - | - | - | - | - | - | - | - | 8.23e-04 | 1 |
| **Bacteroides_faecis** | - | - | - | - | - | - | - | - | - | - | 2.96e-03 | 1 |
| **Bacteroides_fragilis** | - | - | - | - | 2.30e-02 | 1 | 8.96e-04 | 1 | - | - | 1.84e-02 | 1 |
| **Bacteroides**  **_intestinalis** | 4.24e-02 | -1 | - | - | - | - | - | - | - | - | - | - |
| **Bacteroides**  **_massiliensis** | - | - | - | - | - | - | 3.27e-02 | -1 | - | - | 9.19e-04 | 1 |
| **Bacteroides_nordii** | - | - | - | - | - | - | - | - | - | - | 1.60e-02 | 1 |
| **Bacteroides**  **_pectinophilus** | - | - | - | - | - | - | - | - | - | - | 4.24e-03 | -1 |
| **Bacteroides_plebeius** | - | - | - | - | - | - | - | - | - | - | 2.34e-02 | 1 |
| **Bacteroides_salyersiae** | - | - | - | - | - | - | - | - | - | - | 4.82e-03 | 1 |
| **Bacteroides_stercoris** | - | - | - | - | - | - | - | - | 3.87e-02 | 1 | 1.83e-02 | 1 |
| **Bacteroides_vulgatus** | - | - | - | - | - | - | - | - | - | - | 1.28e-02 | -1 |
| **Bacteroides**  **_xylanisolvens** | - | - | - | - | - | - | - | - | - | - | 3.56e-03 | 1 |
| **Barnesiella**  **_intestinihominis** | - | - | - | - | - | - | 1.61e-02 | 1 | - | - | 1.32e-02 | 1 |
| **Bifidobacterium**  **_adolescentis** | 2.73e-02 | -1 | - | - | - | - | - | - | - | - | - | - |
| **Bifidobacterium**  **_angulatum** | - | - | - | - | - | - | - | - | 3.63e-02 | -1 | 2.00e-02 | 1 |
| **Bifidobacterium**  **_animalis** | - | - | - | - | - | - | - | - | - | - | 2.35e-03 | -1 |
| **Bifidobacterium_breve** | - | - | - | - | - | - | - | - | 3.16e-02 | -1 | - | - |
| **Bifidobacterium**  **_catenulatum** | 4.81e-02 | -1 | - | - | - | - | - | - | 4.08e-04 | -1 | - | - |
| **Bifidobacterium**  **_longum** | 4.96e-02 | -1 | - | - | - | - | - | - | 1.61e-02 | -1 | - | - |
| **Bifidobacterium**  **_pseudocatenulatum** | - | - | 4.84e-02 | -1 | - | - | - | - | 5.95e-03 | -1 | - | - |
| **Bilophila_unclassified** | - | - | - | - | - | - | - | - | 7.52e-04 | 1 | 2.88e-02 | 1 |
| **Bilophila_wadsworthia** | - | - | - | - | - | - | - | - | 3.51e-02 | 1 | 3.15e-02 | 1 |
| **Blautia_producta** | - | - | - | - | 1.35e-02 | 1 | 1.54e-02 | 1 | - | - | - | - |
| **Brevibacterium**  **_unclassified** | - | - | - | - | - | - | - | - | - | - | 4.52e-02 | 1 |
| **Burkholderiales_bacterium_1_1_47** | - | - | - | - | - | - | 3.26e-02 | -1 | - | - | - | - |
| **Butyricicoccus**  **_pullicaecorum** | - | - | - | - | - | - | - | - | - | - | 6.93e-03 | 1 |
| **Butyrivibrio_crossotus** | - | - | - | - | - | - | - | - | - | - | 3.82e-02 | 1 |
| **Butyrivibrio**  **_unclassified** | - | - | - | - | - | - | - | - | 1.80e-02 | -1 | - | - |
| **Campylobacter**  **_gracilis** | - | - | - | - | - | - | - | - | 3.45e-02 | 1 | - | - |
| **Campylobacter**  **_ureolyticus** | - | - | - | - | 1.24e-02 | 1 | - | - | 8.35e-03 | 1 | 2.00e-02 | 1 |
| **Candidatus_Zinderia**  **_insecticola** | - | - | - | - | - | - | - | - | - | - | 3.02e-04 | -1 |
| **Catenibacterium**  **_mitsuokai** | - | - | - | - | - | - | 2.28e-02 | -1 | - | - | 2.12e-02 | 1 |
| **Cellulophaga**  **_unclassified** | - | - | - | - | - | - | - | - | 2.00e-02 | -1 | 2.00e-02 | 1 |
| **Chicory_yellow_mottle_virus_large_satellite_RNA** | - | - | - | - | - | - | - | - | - | - | 3.16e-02 | -1 |
| **Citrobacter**  **_unclassified** | - | - | - | - | - | - | - | - | - | - | 1.32e-02 | 1 |
| **Clostridiaceae_bacterium_JC118** | - | - | - | - | - | - | - | - | - | - | 3.68e-02 | -1 |
| **Clostridiales_bacterium_1_7_47FAA** | - | - | - | - | - | - | 1.02e-03 | 1 | - | - | - | - |
| **Clostridium**  **_asparagiforme** | - | - | - | - | - | - | - | - | - | - | 9.60e-03 | 1 |
| **Clostridium_bartlettii** | - | - | - | - | - | - | - | - | 7.70e-03 | -1 | - | - |
| **Clostridium_bolteae** | - | - | - | - | 2.26e-02 | 1 | 5.41e-03 | 1 | - | - | - | - |
| **Clostridium_citroniae** | - | - | - | - | - | - | 1.71e-02 | 1 | 3.73e-02 | 1 | - | - |
| **Clostridium**  **_clostridioforme** | - | - | - | - | 4.41e-02 | 1 | 4.75e-04 | 1 | 1.95e-02 | 1 | - | - |
| **Clostridium**  **_hathewayi** | - | - | - | - | - | - | 8.00e-06 | 1 | 3.79e-05 | 1 | 6.94e-04 | -1 |
| **Clostridium**  **_hylemonae** | - | - | - | - | - | - | - | - | 2.86e-02 | 1 | - | - |
| **Clostridium_nexile** | - | - | - | - | - | - | - | - | - | - | 3.52e-03 | 1 |
| **Clostridium_ramosum** | - | - | - | - | - | - | 2.90e-03 | 1 | - | - | - | - |
| **Clostridium_scindens** | - | - | - | - | - | - | 3.18e-03 | 1 | - | - | - | - |
| **Clostridium_sp_ATCC_BAA_442** | - | - | - | - | - | - | 7.98e-04 | 1 | - | - | - | - |
| **Clostridium_sp_KLE_1755** | - | - | - | - | - | - | - | - | - | - | 4.42e-02 | 1 |
| **Clostridium_sp_L2_50** | - | - | - | - | - | - | 4.19e-02 | -1 | - | - | - | - |
| **Clostridium**  **_symbiosum** | 1.93e-02 | 1 | - | - | 4.37e-03 | 1 | 4.05e-06 | 1 | 2.25e-05 | 1 | 6.21e-03 | -1 |
| **Coprobacillus**  **_unclassified** | - | - | - | - | - | - | 2.17e-03 | 1 | - | - | - | - |
| **Coprobacter**  **_fastidiosus** | - | - | - | - | - | - | - | - | - | - | 1.23e-02 | 1 |
| **Coprococcus_comes** | - | - | - | - | - | - | 1.60e-02 | -1 | - | - | 4.60e-02 | -1 |
| **Coprococcus_eutactus** | - | - | - | - | - | - | 3.08e-02 | 1 | 1.20e-02 | 1 | - | - |
| **Coprococcus**  **_sp_ART55_1** | - | - | - | - | - | - | 1.53e-02 | -1 | - | - | - | - |
| **Corynebacterium**  **_casei** | - | - | - | - | - | - | - | - | - | - | 1.85e-02 | -1 |
| **Dasheen_mosaic_virus** | - | - | - | - | - | - | - | - | - | - | 1.43e-10 | -1 |
| **Desulfovibrio**  **_desulfuricans** | - | - | - | - | - | - | 3.25e-02 | 1 | 2.42e-02 | 1 | 1.95e-02 | 1 |
| **Dialister_invisus** | - | - | - | - | - | - | - | - | - | - | 1.43e-05 | 1 |
| **Dorea**  **_formicigenerans** | - | - | - | - | - | - | 3.30e-02 | 1 | - | - | - | - |
| **Eggerthella_lenta** | - | - | - | - | - | - | 1.59e-02 | 1 | - | - | - | - |
| **Eggerthella_sp_1_3_56FAA** | - | - | - | - | - | - | - | - | 3.06e-02 | 1 | - | - |
| **Eggerthella**  **_unclassified** | - | - | - | - | - | - | 4.26e-02 | 1 | - | - | - | - |
| **Eikenella_corrodens** | 2.02e-02 | 1 | - | - | - | - | 9.39e-03 | 1 | 8.87e-03 | 1 | 2.00e-02 | 1 |
| **Enterobacter_cloacae** | - | - | - | - | 8.52e-03 | -1 | - | - | - | - | - | - |
| **Enterococcus_avium** | - | - | - | - | - | - | 1.47e-02 | 1 | 7.96e-03 | -1 | - | - |
| **Enterococcus_faecalis** | - | - | - | - | - | - | - | - | 3.62e-02 | -1 | - | - |
| **Enterococcus_faecium** | - | - | - | - | - | - | - | - | 1.27e-02 | 1 | - | - |
| **Enterococcus**  **_gallinarum** | - | - | - | - | - | - | - | - | 1.81e-02 | -1 | - | - |
| **Enterococcus_gilvus** | - | - | - | - | - | - | - | - | 4.14e-02 | -1 | - | - |
| **Enterococcus**  **_malodoratus** | - | - | - | - | - | - | - | - | 3.53e-03 | -1 | - | - |
| **Enterorhabdus**  **_caecimuris** | - | - | - | - | - | - | - | - | 4.14e-02 | -1 | - | - |
| **Eremothecium**  **_unclassified** | - | - | - | - | - | - | - | - | 3.45e-02 | 1 | - | - |
| **Erysipelotrichaceae_bacterium_5_2_54FAA** | - | - | - | - | - | - | 3.86e-02 | 1 | 3.86e-02 | -1 | - | - |
| **Escherichia_coli** | 7.53e-03 | 1 | 3.31e-02 | 1 | 7.32e-03 | 1 | - | - | - | - | 7.00e-03 | 1 |
| **Escherichia**  **_unclassified** | - | - | 4.65e-02 | 1 | - | - | - | - | - | - | - | - |
| **Eubacterium_biforme** | - | - | - | - | - | - | - | - | - | - | 4.38e-03 | 1 |
| **Eubacterium_brachy** | - | - | - | - | - | - | - | - | 1.57e-03 | 1 | - | - |
| **Eubacterium_eligens** | 5.29e-03 | -1 | - | - | - | - | 1.28e-03 | -1 | 3.05e-04 | -1 | - | - |
| **Eubacterium_hallii** | 2.44e-02 | -1 | - | - | - | - | 3.83e-02 | -1 | 4.92e-06 | -1 | - | - |
| **Eubacterium**  **_infirmum** | - | - | - | - | - | - | 4.38e-03 | 1 | - | - | - | - |
| **Eubacterium_limosum** | - | - | - | - | - | - | 1.46e-03 | 1 | - | - | - | - |
| **Eubacterium_rectale** | - | - | - | - | - | - | 5.47e-03 | -1 | - | - | - | - |
| **Eubacterium_siraeum** | - | - | - | - | - | - | - | - | 2.92e-02 | 1 | - | - |
| **Eubacterium**  **_sp_3_1_31** | - | - | - | - | - | - | - | - | 2.27e-02 | -1 | - | - |
| **Eubacterium**  **_ventriosum** | - | - | 4.08e-02 | 1 | - | - | 1.84e-04 | -1 | 4.86e-04 | -1 | - | - |
| **Faecalibacterium**  **_prausnitzii** | 4.43e-02 | -1 | - | - | - | - | 5.83e-04 | -1 | - | - | - | - |
| **Flavonifractor_plautii** | - | - | - | - | - | - | - | - | 1.09e-03 | 1 | 5.22e-03 | 1 |
| **Fusobacterium**  **_gonidiaformans** | - | - | - | - | - | - | - | - | 2.11e-02 | 1 | 4.52e-02 | 1 |
| **Fusobacterium**  **_mortiferum** | 3.61e-02 | 1 | - | - | - | - | 3.92e-02 | 1 | - | - | - | - |
| **Fusobacterium**  **_necrophorum** | - | - | - | - | - | - | - | - | - | - | 2.00e-02 | 1 |
| **Fusobacterium_nucleatum** | - | - | 2.44e-02 | 1 | 1.70e-04 | 1 | 1.84e-06 | 1 | 4.48e-07 | 1 | 2.63e-08 | 1 |
| **Fusobacterium**  **_varium** | - | - | - | - | - | - | 2.77e-02 | 1 | - | - | - | - |
| **Gemella_morbillorum** | - | - | 2.09e-05 | 1 | 3.68e-03 | 1 | 6.58e-08 | 1 | 4.73e-04 | 1 | 3.70e-06 | 1 |
| **Gemella_sanguinis** | - | - | - | - | - | - | - | - | 9.80e-03 | -1 | - | - |
| **Gordonibacter**  **_pamelaeae** | 4.39e-03 | -1 | - | - | - | - | - | - | - | - | - | - |
| **Granulicatella**  **_adiacens** | - | - | - | - | - | - | 1.37e-02 | 1 | - | - | - | - |
| **Haemophilus**  **_parainfluenzae** | - | - | - | - | - | - | 1.63e-03 | -1 | - | - | 4.90e-03 | 1 |
| **Haemophilus**  **_sputorum** | - | - | - | - | - | - | 2.79e-03 | -1 | - | - | - | - |
| **Holdemania_filiformis** | 3.72e-02 | 1 | - | - | - | - | - | - | - | - | 3.72e-02 | 1 |
| **Klebsiella_oxytoca** | 2.02e-02 | 1 | - | - | - | - | 4.80e-02 | 1 | - | - | 6.31e-03 | 1 |
| **Klebsiella_unclassified** | - | - | - | - | - | - | - | - | - | - | 5.65e-03 | 1 |
| **Lachnospiraceae_bacterium_2_1_58FAA** | - | - | - | - | - | - | - | - | 3.82e-02 | 1 | - | - |
| **Lachnospiraceae_bacterium_3_1_46FAA** | - | - | - | - | - | - | - | - | 6.93e-03 | 1 | - | - |
| **Lachnospiraceae_bacterium_3_1_57FAA_CT1** | - | - | - | - | 2.52e-02 | 1 | - | - | 4.44e-02 | 1 | 1.57e-02 | 1 |
| **Lachnospiraceae_bacterium_5_1_57FAA** | - | - | - | - | 4.95e-02 | 1 | 8.65e-03 | 1 | - | - | - | - |
| **Lachnospiraceae_bacterium_5_1_63FAA** | - | - | - | - | - | - | - | - | 8.54e-06 | -1 | - | - |
| **Lachnospiraceae_bacterium_7_1_58FAA** | - | - | - | - | - | - | - | - | 2.87e-05 | 1 | 1.44e-03 | 1 |
| **Lachnospiraceae_bacterium_8_1_57FAA** | - | - | - | - | - | - | - | - | - | - | 4.07e-03 | -1 |
| **Lactobacillus**  **_amylovorus** | - | - | - | - | - | - | - | - | - | - | 4.69e-02 | -1 |
| **Lactobacillus_animalis** | - | - | - | - | - | - | - | - | - | - | 4.88e-02 | -1 |
| **Lactobacillus**  **_crispatus** | - | - | 1.75e-02 | 1 | - | - | - | - | - | - | 4.59e-02 | 1 |
| **Lactobacillus**  **_delbrueckii** | - | - | - | - | - | - | - | - | 4.62e-02 | -1 | - | - |
| **Lactobacillus**  **_fermentum** | 1.12e-02 | -1 | - | - | - | - | - | - | - | - | - | - |
| **Lactobacillus_ruminis** | - | - | - | - | - | - | - | - | 6.94e-04 | -1 | - | - |
| **Lactobacillus**  **_salivarius** | - | - | - | - | - | - | - | - | - | - | 1.94e-02 | 1 |
| **Lactobacillus**  **_sanfranciscensis** | - | - | - | - | 4.34e-02 | 1 | - | - | - | - | - | - |
| **Lactococcus**  **_phage_bIL67** | - | - | - | - | - | - | - | - | - | - | 4.52e-02 | 1 |
| **Lactococcus**  **_phage_jm2** | - | - | - | - | - | - | - | - | 1.77e-02 | -1 | - | - |
| **Lactococcus**  **_phage_P680** | - | - | - | - | - | - | - | - | 4.59e-02 | -1 | - | - |
| **Lactococcus**  **_phage_phi7** | - | - | - | - | - | - | - | - | - | - | 2.00e-02 | 1 |
| **Leptotrichia**  **_unclassified** | - | - | - | - | - | - | - | - | 2.06e-02 | 1 | - | - |
| **Leuconostoc_lactis** | - | - | - | - | - | - | - | - | 4.14e-02 | -1 | - | - |
| **Megasphaera_elsdenii** | - | - | - | - | - | - | - | - | - | - | 4.42e-02 | 1 |
| **Megasphaera**  **_micronuciformis** | - | - | - | - | - | - | - | - | 3.45e-02 | 1 | 1.77e-03 | 1 |
| **Methanobrevibacter**  **_smithii** | - | - | 1.10e-03 | 1 | - | - | - | - | - | - | 1.49e-04 | 1 |
| **Methanobrevibacter**  **_unclassified** | - | - | 1.31e-02 | 1 | - | - | - | - | 4.88e-02 | 1 | - | - |
| **Methanosphaera**  **_stadtmanae** | - | - | 4.22e-02 | 1 | - | - | - | - | 1.93e-02 | -1 | - | - |
| **Morganella_morganii** | 2.69e-02 | 1 | - | - | - | - | 3.63e-02 | 1 | 3.45e-02 | 1 | - | - |
| **Odoribacter**  **_splanchnicus** | - | - | 1.40e-02 | 1 | - | - | - | - | - | - | 7.25e-03 | 1 |
| **Odoribacter**  **_unclassified** | - | - | - | - | - | - | 1.59e-02 | 1 | - | - | - | - |
| **Oscillibacter**  **_unclassified** | - | - | - | - | - | - | - | - | 1.40e-03 | 1 | 8.96e-04 | 1 |
| **Parabacteroides**  **_goldsteinii** | - | - | - | - | - | - | - | - | 2.65e-02 | -1 | - | - |
| **Parabacteroides**  **_merdae** | - | - | - | - | - | - | - | - | 4.76e-03 | 1 | 1.26e-03 | 1 |
| **Parabacteroides**  **_unclassified** | - | - | - | - | - | - | 3.72e-03 | 1 | - | - | - | - |
| **Paraprevotella**  **_unclassified** | - | - | - | - | - | - | - | - | - | - | 2.55e-02 | 1 |
| **Parasutterella**  **_excrementihominis** | - | - | - | - | - | - | 1.46e-02 | -1 | - | - | - | - |
| **Parvimonas_micra** | - | - | 2.09e-05 | 1 | - | - | 1.56e-07 | 1 | 3.95e-04 | 1 | 4.83e-06 | 1 |
| **Parvimonas**  **_unclassified** | - | - | 9.50e-06 | 1 | 2.66e-03 | 1 | 1.97e-09 | 1 | 3.50e-03 | 1 | 2.18e-07 | 1 |
| **Pediococcus**  **_acidilactici** | - | - | - | - | - | - | - | - | 7.93e-03 | -1 | - | - |
| **Pediococcus_lolii** | - | - | - | - | - | - | - | - | 4.14e-02 | -1 | - | - |
| **Peptoniphilus**  **_duerdenii** | - | - | - | - | - | - | - | - | - | - | 4.52e-02 | 1 |
| **Peptostreptococcaceae_noname_unclassified** | - | - | 9.82e-03 | 1 | - | - | 1.21e-02 | 1 | - | - | - | - |
| **Peptostreptococcus**  **_anaerobius** | - | - | - | - | 4.73e-02 | 1 | 2.06e-03 | 1 | - | - | 2.68e-02 | 1 |
| **Peptostreptococcus**  **_stomatis** | - | - | 4.00e-04 | 1 | 4.70e-03 | 1 | 8.43e-10 | 1 | 7.04e-06 | 1 | 2.56e-05 | 1 |
| **Peptostreptococcus**  **_unclassified** | - | - | - | - | - | - | 2.07e-04 | 1 | - | - | - | - |
| **Porphyromonas**  **_asaccharolytica** | 3.32e-03 | 1 | - | - | 5.31e-03 | 1 | 2.83e-04 | 1 | 1.67e-06 | 1 | 4.15e-08 | 1 |
| **Porphyromonas**  **_somerae** | - | - | - | - | - | - | - | - | 9.01e-04 | 1 | 1.13e-05 | 1 |
| **Porphyromonas**  **_uenonis** | - | - | - | - | 9.73e-04 | 1 | - | - | 1.45e-03 | 1 | 4.66e-06 | 1 |
| **Prevotella_copri** | - | - | - | - | - | - | - | - | - | - | 2.05e-09 | 1 |
| **Prevotella_denticola** | - | - | - | - | - | - | - | - | - | - | 4.52e-02 | 1 |
| **Prevotella_intermedia** | - | - | - | - | 4.34e-02 | 1 | 3.42e-03 | 1 | 1.39e-02 | 1 | 8.91e-03 | 1 |
| **Prevotella_nigrescens** | - | - | - | - | - | - | - | - | 3.45e-02 | 1 | - | - |
| **Prevotella_stercorea** | 3.61e-02 | 1 | - | - | - | - | 4.45e-02 | 1 | - | - | - | - |
| **Propionibacterium**  **_acnes** | - | - | - | - | - | - | - | - | 7.20e-03 | -1 | - | - |
| **Pseudoflavonifractor**  **_capillosus** | - | - | - | - | - | - | - | - | 2.22e-03 | 1 | 7.15e-04 | 1 |
| **Pseudomonas_fragi** | - | - | - | - | 4.34e-02 | -1 | - | - | 1.81e-02 | -1 | - | - |
| **Pseudomonas**  **_unclassified** | - | - | - | - | - | - | - | - | - | - | 3.86e-03 | -1 |
| **Roseburia_hominis** | - | - | - | - | 4.52e-02 | -1 | - | - | - | - | - | - |
| **Roseburia_intestinalis** | - | - | - | - | - | - | 4.72e-03 | -1 | 1.19e-02 | -1 | - | - |
| **Rothia_dentocariosa** | - | - | - | - | - | - | 3.24e-02 | 1 | - | - | 3.94e-03 | -1 |
| **Rothia_mucilaginosa** | - | - | - | - | - | - | 6.07e-03 | 1 | - | - | - | - |
| **Ruminococcaceae**  **_bacterium_D16** | - | - | - | - | - | - | - | - | 2.51e-02 | 1 | 1.12e-06 | 1 |
| **Ruminococcus_albus** | - | - | - | - | - | - | 3.71e-04 | -1 | - | - | - | - |
| **Ruminococcus_bromii** | - | - | - | - | - | - | - | - | 2.87e-03 | -1 | - | - |
| **Ruminococcus**  **_champanellensis** | - | - | - | - | - | - | - | - | 1.49e-02 | -1 | - | - |
| **Ruminococcus**  **_flavefaciens** | - | - | - | - | - | - | 2.01e-02 | -1 | 3.29e-02 | -1 | - | - |
| **Ruminococcus_gnavus** | - | - | - | - | 1.49e-02 | 1 | - | - | - | - | - | - |
| **Ruminococcus_lactaris** | - | - | - | - | 4.48e-02 | -1 | - | - | - | - | - | - |
| **Ruminococcus_obeum** | - | - | - | - | - | - | - | - | 2.27e-02 | -1 | - | - |
| **Ruminococcus_sp_5_1_39BFAA** | - | - | - | - | - | - | - | - | 3.50e-04 | -1 | 3.47e-03 | -1 |
| **Ruminococcus_torques** | - | - | - | - | 1.29e-02 | 1 | - | - | - | - | - | - |
| **Shigella_sonnei** | - | - | - | - | - | - | - | - | - | - | 7.79e-04 | 1 |
| **Slackia_exigua** | - | - | - | - | - | - | - | - | 3.45e-02 | 1 | - | - |
| **Slackia_unclassified** | - | - | - | - | - | - | 2.15e-02 | 1 | - | - | - | - |
| **Solobacterium_moorei** | - | - | 4.59e-03 | 1 | 3.98e-02 | 1 | 3.70e-06 | 1 | 1.43e-03 | 1 | 1.26e-02 | 1 |
| **Streptococcus**  **_anginosus** | - | - | - | - | - | - | 8.30e-03 | 1 | - | - | - | - |
| **Streptococcus**  **_australis** | - | - | - | - | - | - | 2.21e-02 | -1 | 8.14e-04 | -1 | - | - |
| **Streptococcus**  **_constellatus** | - | - | 1.20e-02 | 1 | 4.26e-02 | 1 | 3.53e-03 | 1 | - | - | 3.61e-02 | 1 |
| **Streptococcus**  **_dysgalactiae** | - | - | - | - | - | - | - | - | - | - | 4.52e-02 | 1 |
| **Streptococcus**  **_gallolyticus** | - | - | - | - | 2.31e-02 | 1 | - | - | - | - | - | - |
| **Streptococcus_infantis** | - | - | - | - | - | - | - | - | 1.02e-03 | -1 | - | - |
| **Streptococcus**  **_intermedius** | - | - | - | - | - | - | - | - | 1.55e-02 | 1 | - | - |
| **Streptococcus**  **_lutetiensis** | - | - | - | - | - | - | - | - | - | - | 4.52e-02 | 1 |
| **Streptococcus**  **_pasteurianus** | - | - | - | - | - | - | 2.62e-02 | 1 | - | - | - | - |
| **Streptococcus**  **_salivarius** | 3.91e-02 | -1 | - | - | - | - | - | - | 1.00e-05 | -1 | - | - |
| **Streptococcus**  **_sanguinis** | - | - | - | - | - | - | - | - | 2.99e-04 | -1 | 1.70e-02 | -1 |
| **Streptococcus**  **_thermophilus** | - | - | - | - | - | - | - | - | 1.46e-02 | -1 | 2.05e-03 | -1 |
| **Streptococcus**  **_tigurinus** | - | - | - | - | - | - | 5.42e-03 | 1 | - | - | - | - |
| **Streptococcus**  **_vestibularis** | - | - | - | - | - | - | - | - | 3.10e-03 | -1 | - | - |
| **Subdoligranulum_sp_4_3_54A2FAA** | - | - | - | - | - | - | 2.35e-02 | 1 | 2.70e-02 | 1 | - | - |
| **Subdoligranulum**  **_unclassified** | 4.25e-02 | -1 | - | - | - | - | - | - | 2.66e-02 | 1 | - | - |
| **Sutterella**  **_wadsworthensis** | - | - | - | - | - | - | - | - | - | - | 6.19e-04 | 1 |
| **Synergistes_sp_3_1_syn1** | - | - | - | - | - | - | 9.39e-03 | 1 | - | - | - | - |
| **Veillonella_atypica** | - | - | - | - | - | - | - | - | - | - | 3.85e-02 | 1 |
| **Veillonella_dispar** | - | - | - | - | - | - | - | - | - | - | 3.19e-03 | 1 |
| **Vicia_cryptic_virus** | - | - | - | - | - | - | - | - | - | - | 4.96e-05 | -1 |

Note: 1: Upregulated; -1: Downregulated.

**Table S6. Differential species between CRC and CA in three metagenomic datasets.**

| **Species** | **ThomasAM_2018a** | | **ZellerG** | | **FengQ** | |
| --- | --- | --- | --- | --- | --- | --- |
|  | ***P-value*** | **Dir** | ***P-value*** | **Dir** | ***P-value*** | **Dir** |
| **Acidaminococcus_fermentans** | - | - | - | - | 4.10e-02 | 1 |
| **Acidaminococcus_unclassified** | - | - | - | - | 2.35e-04 | 1 |
| **Actinomyces_sp_ICM47** | - | - | - | - | 2.34e-02 | -1 |
| **Alistipes_finegoldii** | - | - | - | - | 2.12e-03 | 1 |
| **Alistipes_onderdonkii** | - | - | - | - | 2.11e-02 | 1 |
| **Alistipes_putredinis** | - | - | - | - | 5.38e-04 | 1 |
| **Alistipes_sp_AP11** | - | - | - | - | 3.14e-02 | 1 |
| **Alloprevotella_tannerae** | 4.95e-02 | 1 | - | - | - | - |
| **A-erococcus_obesiensis** | - | - | 3.61e-02 | 1 | 2.14e-02 | 1 |
| **A-erococcus_vagi-lis** | - | - | - | - | 3.29e-03 | 1 |
| **A-erotruncus_unclassified** | - | - | - | - | 1.62e-02 | 1 |
| **Bacteroides_coprophilus** | - | - | - | - | 3.60e-03 | 1 |
| **Bacteroides_eggerthii** | - | - | - | - | 2.35e-03 | 1 |
| **Bacteroides_faecis** | - | - | - | - | 2.60e-03 | 1 |
| **Bacteroides_fluxus** | - | - | 1.99e-02 | 1 | - | - |
| **Bacteroides_fragilis** | - | - | - | - | 3.44e-02 | -1 |
| **Bacteroides_nordii** | - | - | 4.47e-02 | 1 | - | - |
| **Bacteroides_ovatus** | - | - | - | - | 1.92e-02 | 1 |
| **Bacteroides_pectinophilus** | - | - | 4.52e-02 | -1 | - | - |
| **Bacteroides_stercoris** | - | - | - | - | 3.42e-02 | -1 |
| **Bacteroides_vulgatus** | - | - | - | - | 3.29e-03 | 1 |
| **Bacteroides_xylanisolvens** | - | - | - | - | 1.22e-05 | 1 |
| **Barnesiella_intestinihominis** | - | - | - | - | 6.65e-04 | 1 |
| **Bartonella_unclassified** | - | - | 3.77e-02 | -1 | - | - |
| **Bifidobacterium_breve** | - | - | - | - | 2.86e-02 | 1 |
| **Bifidobacterium_catenulatum** | 2.23e-02 | -1 | - | - | - | - |
| **Bombyx_mori_nucleopolyhedrovirus** | - | - | - | - | 4.10e-02 | 1 |
| **Burkholderia_unclassified** | - | - | - | - | 1.50e-02 | 1 |
| **Burkholderiales_bacterium_1_1_47** | - | - | - | - | 5.97e-03 | 1 |
| **Butyricicoccus_pullicaecorum** | - | - | - | - | 1.37e-02 | 1 |
| **Candidatus_Zinderia_insecticola** | - | - | - | - | 3.98e-02 | -1 |
| **Catenibacterium_mitsuokai** | - | - | - | - | 3.46e-02 | 1 |
| **Cellulophaga_unclassified** | - | - | - | - | 4.10e-02 | 1 |
| **Citrobacter_unclassified** | - | - | - | - | 1.42e-02 | 1 |
| **Clostridium_citroniae** | - | - | - | - | 1.52e-02 | -1 |
| **Clostridium_hathewayi** | - | - | 8.51e-03 | 1 | 1.55e-02 | -1 |
| **Clostridium_symbiosum** | - | - | 7.98e-04 | 1 | 1.20e-03 | -1 |
| **Collinsella_aerofaciens** | 3.27e-02 | -1 | - | - | - | - |
| **Collinsella_intesti-lis** | - | - | 1.87e-02 | -1 | - | - |
| **Collinsella_stercoris** | - | - | 3.77e-02 | -1 | - | - |
| **Coprobacillus_unclassified** | - | - | 4.64e-02 | 1 | - | - |
| **Coprobacter_fastidiosus** | - | - | - | - | 1.35e-02 | 1 |
| **Coprococcus_comes** | - | - | 4.47e-02 | -1 | 3.33e-02 | -1 |
| **Dasheen_mosaic_virus** | - | - | - | - | 5.33e-08 | -1 |
| **Desulfovibrio_piger** | 4.45e-02 | 1 | - | - | - | - |
| **Dialister_invisus** | - | - | - | - | 3.87e-02 | 1 |
| **Dialister_succi-tiphilus** | - | - | - | - | 4.94e-02 | -1 |
| **Dorea_longicate-** | - | - | 1.21e-02 | -1 | - | - |
| **Eggerthella_sp_HGA1** | - | - | - | - | 4.55e-02 | -1 |
| **Eikenella_corrodens** | - | - | 3.61e-02 | 1 | 4.10e-02 | 1 |
| **Enterobacteriaceae_bacterium_9_2_54FAA** | - | - | 1.83e-02 | -1 | - | - |
| **Enterococcus_avium** | 4.95e-02 | 1 | - | - | - | - |
| **Enterococcus_durans** | - | - | 2.55e-02 | 1 | - | - |
| **Enterococcus_faecalis** | - | - | 3.33e-02 | -1 | - | - |
| **Enterococcus_gilvus** | - | - | 3.77e-02 | -1 | - | - |
| **Enterococcus_malodoratus** | - | - | 2.98e-03 | -1 | - | - |
| **Escherichia_coli** | - | - | - | - | 4.27e-03 | 1 |
| **Eubacterium_infirmum** | - | - | - | - | 2.94e-02 | -1 |
| **Eubacterium_rectale** | - | - | 8.16e-03 | -1 | - | - |
| **Eubacterium_ventriosum** | - | - | 1.15e-03 | -1 | - | - |
| **Fusobacterium_gonidiaformans** | - | - | 2.66e-02 | 1 | - | - |
| **Fusobacterium_necrophorum** | - | - | - | - | 4.10e-02 | 1 |
| **Fusobacterium_nucleatum** | - | - | 1.46e-05 | 1 | 4.21e-05 | 1 |
| **Gemella_morbillorum** | - | - | 1.31e-04 | 1 | 3.38e-05 | 1 |
| **Granulicatella_unclassified** | - | - | - | - | 4.85e-02 | -1 |
| **Haemophilus_parainfluenzae** | - | - | - | - | 3.47e-02 | 1 |
| **Hafnia_alvei** | - | - | 8.27e-03 | -1 | - | - |
| **Holdemania_filiformis** | - | - | - | - | 6.99e-03 | 1 |
| **Klebsiella_oxytoca** | - | - | - | - | 4.26e-03 | 1 |
| **Klebsiella_unclassified** | - | - | - | - | 2.90e-03 | 1 |
| **Lachnospiraceae_bacterium_1_1_57FAA** | - | - | 1.46e-02 | -1 | - | - |
| **Lachnospiraceae_bacterium_2_1_58FAA** | - | - | 3.77e-02 | 1 | - | - |
| **Lachnospiraceae_bacterium_7_1_58FAA** | - | - | - | - | 9.84e-03 | 1 |
| **Lachnospiraceae_bacterium_8_1_57FAA** | - | - | - | - | 5.72e-03 | -1 |
| **Lactobacillus_acidophilus** | - | - | - | - | 4.55e-02 | -1 |
| **Lactobacillus_brevis** | - | - | 1.05e-02 | -1 | - | - |
| **Lactobacillus_curvatus** | - | - | - | - | 1.52e-02 | 1 |
| **Lactobacillus_fermentum** | 3.47e-02 | -1 | - | - | - | - |
| **Lactobacillus_johnsonii** | - | - | 1.91e-02 | -1 | 4.10e-02 | 1 |
| **Lactobacillus_plantarum** | - | - | 3.41e-02 | -1 | - | - |
| **Lactobacillus_ruminis** | - | - | 2.98e-03 | -1 | - | - |
| **Lactobacillus_saerimneri** | - | - | 3.77e-02 | -1 | - | - |
| **Lactobacillus_salivarius** | - | - | - | - | 7.87e-03 | 1 |
| **Lactococcus_phage_P680** | - | - | 9.22e-03 | -1 | 2.53e-03 | 1 |
| **Lactococcus_phage_phi7** | - | - | - | - | 4.10e-02 | 1 |
| **Lactococcus_phage_ul36** | - | - | 3.77e-02 | -1 | - | - |
| **Leptotrichia_unclassified** | - | - | 2.66e-02 | 1 | - | - |
| **Morganella_morganii** | 3.77e-03 | 1 | - | - | - | - |
| **Neisseria_unclassified** | 4.95e-02 | 1 | - | - | - | - |
| **Odoribacter_splanchnicus** | 3.10e-02 | 1 | - | - | - | - |
| **Odoribacter_unclassified** | 2.46e-02 | -1 | - | - | - | - |
| **Oscillibacter_unclassified** | - | - | - | - | 2.00e-02 | 1 |
| **Parabacteroides_distasonis** | - | - | - | - | 1.43e-02 | 1 |
| **Parabacteroides_merdae** | - | - | - | - | 2.28e-04 | 1 |
| **Parasutterella_excrementihominis** | - | - | - | - | 9.51e-03 | 1 |
| **Parvimo-s_micra** | - | - | 1.85e-04 | 1 | 6.22e-04 | 1 |
| **Parvimo-s_unclassified** | - | - | 9.24e-04 | 1 | 1.92e-04 | 1 |
| **Pediococcus_lolii** | - | - | 1.05e-02 | -1 | - | - |
| **Pediococcus_unclassified** | - | - | - | - | 4.55e-02 | -1 |
| **Peptostreptococcus_a-erobius** | - | - | - | - | 3.38e-02 | 1 |
| **Peptostreptococcus_stomatis** | - | - | 5.11e-06 | 1 | 1.70e-02 | 1 |
| **Peptostreptococcus_unclassified** | 1.40e-02 | 1 | - | - | - | - |
| **Porphyromo-s_asaccharolytica** | 1.06e-02 | 1 | 4.28e-05 | 1 | 7.89e-04 | 1 |
| **Porphyromo-s_somerae** | - | - | 7.65e-03 | 1 | 8.74e-03 | 1 |
| **Porphyromo-s_uenonis** | - | - | 4.05e-03 | 1 | 7.49e-04 | 1 |
| **Prevotella_copri** | - | - | 3.13e-02 | -1 | 1.00e-05 | 1 |
| **Prevotella_intermedia** | - | - | 4.91e-02 | 1 | 2.14e-02 | 1 |
| **Prevotella_timonensis** | - | - | - | - | 4.55e-02 | -1 |
| **Propionibacterium_freudenreichii** | - | - | - | - | 4.89e-02 | -1 |
| **Pseudoflavonifractor_capillosus** | - | - | - | - | 1.14e-02 | 1 |
| **Pseudomo-s_aeruginosa** | 4.28e-02 | 1 | - | - | - | - |
| **Raoultella_ornithinolytica** | 4.95e-02 | 1 | - | - | - | - |
| **Roseburia_intesti-lis** | - | - | 3.21e-02 | 1 | - | - |
| **Roseburia_unclassified** | - | - | - | - | 4.62e-02 | -1 |
| **Rothia_unclassified** | - | - | 2.29e-02 | 1 | - | - |
| **Ruminococcaceae_bacterium_D16** | - | - | - | - | 7.35e-04 | 1 |
| **Ruminococcus_callidus** | - | - | 3.96e-02 | -1 | - | - |
| **Ruminococcus_lactaris** | - | - | 2.76e-02 | -1 | - | - |
| **Salmonella_phage_HK620** | - | - | 1.99e-02 | 1 | - | - |
| **Selenomo-s_bovis** | - | - | 3.77e-02 | -1 | - | - |
| **Shigella_sonnei** | - | - | - | - | 1.49e-02 | 1 |
| **Solobacterium_moorei** | - | - | 2.64e-03 | 1 | - | - |
| **Streptococcus_anginosus** | - | - | 1.81e-02 | 1 | - | - |
| **Streptococcus_australis** | - | - | 3.94e-02 | -1 | 7.51e-03 | -1 |
| **Streptococcus_salivarius** | - | - | 7.08e-04 | -1 | - | - |
| **Streptococcus_tigurinus** | - | - | - | - | 5.50e-03 | 1 |
| **Subdoligranulum_sp_4_3_54A2FAA** | - | - | 2.39e-02 | 1 | - | - |
| **Vicia_cryptic_virus** | - | - | - | - | 5.17e-06 | -1 |
| **Weissella_unclassified** | - | - | 1.84e-02 | -1 | - | - |

Note: 1: Upregulated; -1: Downregulated.

**Table S7. Differential genera between CRC and HC in mixed metagenomic dataset.**

| **Genus** | *p* | Dir | FDR |
| --- | --- | --- | --- |
| Acidaminococcus | 8.89E-03 | 1 | 5.15E-02 |
| Aeromonas | 1.54E-04 | 1 | 1.94E-03 |
| Aggregatibacter | 9.20E-03 | 1 | 5.22E-02 |
| Alistipes | 9.54E-03 | 1 | 5.31E-02 |
| Alloprevotella | 3.59E-03 | 1 | 2.44E-02 |
| Eubacterium | 5.95E-09 | -1 | 2.76E-07 |
| Anaerococcus^a^ | 3.25E-07 | 1 | 1.13E-05 |
| Anaeroglobus | 3.78E-02 | 1 | 1.52E-01 |
| Anaerotruncus | 1.02E-04 | 1 | 1.42E-03 |
| Atopobium | 1.87E-03 | 1 | 1.48E-02 |
| Bilophila | 2.20E-04 | 1 | 2.44E-03 |
| Candidatus_Zinderia | 1.78E-05 | -1 | 3.97E-04 |
| Butyricimonas | 8.76E-03 | 1 | 5.15E-02 |
| Campylobacter | 7.71E-06 | 1 | 2.14E-04 |
| Ruminococcus | 3.09E-05 | -1 | 5.73E-04 |
| Clostridiaceae_noname | 3.99E-02 | 1 | 1.58E-01 |
| Anaerostipes^a^ | 5.41E-05 | -1 | 8.85E-04 |
| Alphacryptovirus | 6.34E-05 | -1 | 9.79E-04 |
| Bifidobacterium | 1.01E-04 | -1 | 1.42E-03 |
| Clostridiales_Family_XIII_Incertae_Sedis_unclassified | 1.09E-02 | 1 | 5.93E-02 |
| Clostridium | 1.25E-04 | 1 | 1.66E-03 |
| Coprobacillus | 3.58E-02 | 1 | 1.49E-01 |
| Desulfovibrio | 1.39E-03 | 1 | 1.14E-02 |
| Faecalibacterium | 2.01E-04 | -1 | 2.33E-03 |
| Eikenella^a^ | 5.80E-08 | 1 | 2.30E-06 |
| Eremothecium | 2.16E-02 | 1 | 9.99E-02 |
| Escherichia^a^ | 2.18E-05 | 1 | 4.32E-04 |
| Coprococcus | 6.84E-04 | -1 | 6.79E-03 |
| Potyvirus | 9.76E-04 | -1 | 9.35E-03 |
| Gordonibacter | 1.05E-03 | -1 | 9.74E-03 |
| Filifactor | 2.91E-03 | 1 | 2.07E-02 |
| Viruses_noname | 1.15E-03 | -1 | 9.98E-03 |
| Flavonifractor | 1.32E-03 | 1 | 1.11E-02 |
| Fretibacterium | 3.55E-02 | 1 | 1.49E-01 |
| Fusobacterium^a^ | 5.80E-25 | 1 | 8.06E-23 |
| Adlercreutzia | 1.91E-03 | -1 | 1.48E-02 |
| Gemella^a^ | 1.70E-05 | 1 | 3.97E-04 |
| Klebsiella | 6.36E-03 | 1 | 3.84E-02 |
| Leptotrichia | 1.65E-04 | 1 | 1.99E-03 |
| Leptotrichiaceae_unclassified | 4.99E-05 | 1 | 8.66E-04 |
| Megasphaera | 4.38E-03 | 1 | 2.77E-02 |
| Streptococcus | 3.68E-03 | -1 | 2.44E-02 |
| Methanobrevibacter | 2.05E-03 | 1 | 1.54E-02 |
| Morganella^a^ | 1.86E-05 | 1 | 3.97E-04 |
| Roseburia | 4.84E-03 | -1 | 2.99E-02 |
| Odoribacter | 1.18E-02 | 1 | 6.20E-02 |
| Oscillibacter | 1.09E-03 | 1 | 9.74E-03 |
| Parabacteroides | 1.33E-02 | 1 | 6.73E-02 |
| Parascardovia | 3.19E-02 | 1 | 1.39E-01 |
| Parvimonas^a^ | 3.36E-21 | 1 | 3.11E-19 |
| Peptoniphilus | 4.04E-02 | 1 | 1.58E-01 |
| Pseudomonas | 1.18E-02 | -1 | 6.20E-02 |
| Peptostreptococcus^a^ | 1.51E-25 | 1 | 4.21E-23 |
| Rothia | 1.24E-02 | -1 | 6.37E-02 |
| Porphyromonas^a^ | 1.81E-19 | 1 | 1.26E-17 |
| Prevotella | 4.17E-06 | 1 | 1.29E-04 |
| Proteus | 3.47E-03 | 1 | 2.41E-02 |
| Retroviridae_noname | 1.65E-02 | -1 | 7.89E-02 |
| Catenibacterium | 1.73E-02 | -1 | 8.17E-02 |
| Pseudoflavonifractor | 3.79E-03 | 1 | 2.45E-02 |
| Actinobacillus | 2.47E-02 | -1 | 1.12E-01 |
| Clostridiales_noname | 2.54E-02 | -1 | 1.12E-01 |
| Bartonella | 2.55E-02 | -1 | 1.12E-01 |
| Ruminococcaceae_noname | 3.54E-04 | 1 | 3.65E-03 |
| Slackia | 3.24E-02 | -1 | 1.39E-01 |
| Selenomonas | 1.59E-02 | 1 | 7.76E-02 |
| Shigella | 1.52E-02 | 1 | 7.54E-02 |
| Solobacterium^a^ | 2.76E-11 | 1 | 1.54E-09 |
| Rhodococcus | 3.78E-02 | -1 | 1.52E-01 |
| Synergistes | 2.55E-04 | 1 | 2.72E-03 |
| Treponema | 2.91E-03 | 1 | 2.07E-02 |

Note: 1: Upregulated; -1: Downregulated; a: the genera with consist direction of dysregulation in multiple analyses.

**Table S8. Differential genera between CRC and CA in mixed metagenomic dataset.**

| **Genus** | *p* | Dir | FDR |
| --- | --- | --- | --- |
| Acidaminococcus | 5.90E-03 | 1 | 5.07E-02 |
| Actinobaculum | 2.72E-02 | -1 | 1.50E-01 |
| Adlercreutzia | 6.66E-03 | -1 | 5.35E-02 |
| Aeromonas | 1.51E-02 | 1 | 9.47E-02 |
| Aggregatibacter | 3.88E-02 | -1 | 1.92E-01 |
| Alistipes | 3.62E-02 | 1 | 1.86E-01 |
| Alloprevotella | 3.61E-03 | 1 | 3.87E-02 |
| Alphacryptovirus | 6.30E-11 | -1 | 5.40E-09 |
| Anaerococcus | 1.03E-03 | 1 | 1.65E-02 |
| Anaerotruncus | 6.47E-03 | 1 | 5.35E-02 |
| Bartonella | 9.71E-05 | -1 | 2.27E-03 |
| Bilophila | 3.01E-03 | -1 | 3.50E-02 |
| Butyricimonas | 1.28E-03 | 1 | 1.65E-02 |
| Campylobacter | 5.65E-03 | 1 | 5.07E-02 |
| Candidatus_Saccharibacteria_noname | 4.43E-02 | 1 | 2.08E-01 |
| Candidatus_Zinderia | 5.73E-06 | -1 | 2.10E-04 |
| Clostridiaceae_noname | 4.96E-02 | 1 | 2.23E-01 |
| Clostridiales_noname | 1.35E-03 | 1 | 1.65E-02 |
| Clostridium | 4.44E-02 | 1 | 2.08E-01 |
| Collinsella | 1.51E-02 | -1 | 9.47E-02 |
| Coprobacillus | 1.28E-03 | 1 | 1.65E-02 |
| Coprococcus | 5.77E-04 | -1 | 1.06E-02 |
| Dermatophilaceae_unclassified | 1.20E-02 | -1 | 8.09E-02 |
| Eikenella | 3.49E-02 | 1 | 1.83E-01 |
| Enterobacteriaceae_noname | 3.25E-02 | -1 | 1.74E-01 |
| Escherichia | 8.43E-03 | 1 | 6.13E-02 |
| Eubacterium | 5.12E-03 | -1 | 5.07E-02 |
| Faecalibacterium | 1.35E-03 | -1 | 1.65E-02 |
| Flavonifractor | 7.66E-03 | 1 | 5.96E-02 |
| Fusobacterium | 2.17E-13 | 1 | 4.01E-11 |
| Gemella | 6.00E-05 | 1 | 1.54E-03 |
| Hafnia | 8.41E-03 | -1 | 6.13E-02 |
| Klebsiella | 7.95E-04 | 1 | 1.36E-02 |
| Lachnospiraceae_noname | 1.39E-02 | -1 | 9.16E-02 |
| Leptotrichia | 5.91E-03 | 1 | 5.07E-02 |
| Leptotrichiaceae_unclassified | 8.59E-03 | 1 | 6.13E-02 |
| Megamonas | 4.65E-02 | 1 | 2.13E-01 |
| Megasphaera | 3.94E-04 | -1 | 7.79E-03 |
| Morganella | 5.83E-03 | 1 | 5.07E-02 |
| Odoribacter | 5.92E-03 | 1 | 5.07E-02 |
| Orthohepadnavirus | 1.80E-02 | -1 | 1.05E-01 |
| Oscillibacter | 3.69E-02 | 1 | 1.86E-01 |
| Parabacteroides^a^ | 1.96E-05 | 1 | 6.28E-04 |
| Parvimonas^a^ | 3.70E-09 | 1 | 1.90E-07 |
| Peptostreptococcus^a^ | 3.12E-13 | 1 | 4.01E-11 |
| Porphyromonas^a^ | 3.09E-09 | 1 | 1.90E-07 |
| Potyvirus | 1.74E-04 | -1 | 3.72E-03 |
| Prevotella | 3.13E-03 | 1 | 3.50E-02 |
| Propionibacterium | 1.02E-06 | -1 | 4.39E-05 |
| Pseudoflavonifractor | 2.75E-02 | 1 | 1.50E-01 |
| Ralstonia | 1.80E-02 | -1 | 1.05E-01 |
| Rhodococcus | 2.72E-02 | -1 | 1.50E-01 |
| Saccharomyces | 4.15E-02 | -1 | 2.01E-01 |
| Solobacterium | 3.07E-05 | 1 | 8.76E-04 |
| Synergistes | 9.79E-03 | 1 | 6.80E-02 |
| Tetragenococcus | 1.80E-02 | -1 | 1.05E-01 |
| Viruses_noname | 1.13E-03 | -1 | 1.65E-02 |

Note: 1: Upregulated; -1: Downregulated; a: the genera with consist direction of dysregulation in multiple analyses.

**Table S9. Consistently dysregulated pathways in CRC compared to HC and CA groups**

| **Cohorts** | **Variable** | **L-lysine fermentation to acetate and butanoate** | **pyruvate fermentation to butanoate** | **2-methylbutanoate biosynthesis** | **acetyl-CoA fermentation to butanoate** |
| --- | --- | --- | --- | --- | --- |
| **FengQ** | ***P*** | 2.37e-04 | 0.06 | 0.13 | 0.49 |
|  | **Dir** | 1 | 1 | -1 | 1 |
|  | **Mean (SE)** | 7.25e-07 (2.85e-06) | 3.18e-05 (2.19e-05) | 7.43e-09 (5.33e-08) | 7.81e-05 (5.22e-05) |
| **ThomasAM_2018a** | ***P*** | 0.37 | 0.57 | - | 0.28 |
|  | **Dir** | 1 | -1 | - | 1 |
|  | **Mean (SE)** | 3.99e-07 (2.23e-06) | 1.72e-05 (1.45e-05) | - | 4.33e-05 (2.46e-05) |
| **ThomasAM_2018b** | ***P*** | 6.69e-03 | 0.17 | - | 0.27 |
|  | **Dir** | -1 | -1 | - | 1 |
|  | **Mean (SE)** | 5.59e-07 (1.67e-06) | 1.92e-05 (1.09e-05) | - | 5.85e-05 (2.36e-05) |
| **VogtmannE** | ***P*** | 0.18 | 0.81 | - | 0.21 |
|  | **Dir** | 1 | 1 | - | 1 |
|  | **Mean (SE)** | 2.18e-06 (3.84e-06) | 3.84e-05 (2.42e-05) | - | 6.11e-05 (3.18e-05) |
| **YuJ** | ***P*** | 1.77e-06 | 0.44 | - | 0.33 |
|  | **Dir** | 1 | -1 | - | 1 |
|  | **Mean (SE)** | 2.22e-06 (4.53e-06) | 3.91e-05 (2.27e-05) | - | 8.21e-05 (5.36e-05) |
| **ZellerG** | ***P*** | 4.14e-09 | 0.48 | 0.40 | 0.95 |
|  | **Dir** | 1 | -1 | 1 | 1 |
|  | **Mean (SE)** | 1.73e-06 (4.02e-06) | 3.66e-05 (1.98e-05) | 5.17e-09 (5.14e-08) | 6.46e-05 (3.58e-05) |

Note: 1: Upregulated; -1: Downregulated.
